# Understanding a Substrate’s Product Regioselectivity in a Family of Enzymes: A Case Study of Acetaminophen Binding in Cytochrome P450s

**DOI:** 10.1371/journal.pone.0087058

**Published:** 2014-02-03

**Authors:** Yue Yang, Sergio E. Wong, Felice C. Lightstone

**Affiliations:** Biosciences and Biotechnology Division, Lawrence Livermore National Laboratory, Livermore, California, United States of America; Monash University, Australia

## Abstract

Product regioselectivity as influenced by molecular recognition is a key aspect of enzyme catalysis. We applied large-scale two-dimensional (2D) umbrella sampling (USP) simulations to characterize acetaminophen (APAP) binding in the active sites of the family of Cytochrome P450 (CYP) enzymes as a case study to show the different regioselectivity exhibited by a single substrate in comparative enzymes. Our results successfully explain the experimentally observed product regioselectivity for all five human CYPs included in this study, demonstrating that binding events play an important role in determining regioselectivity. In CYP2C9 and CYP3A4, weak interactions in an overall large active site cavity result in a fairly small binding free energy difference between APAP reactive binding states, consistent with experimental results that show little preference for resulting metabolites. In contrast, in CYP1A2 and CYP2E1, APAP is strongly restrained by a compact binding pocket, leading to a preferred binding conformation. The calculated binding equilibrium of APAP within the compact active site of CYP2A6 is able to predict the experimentally documented product ratios and is also applied to explain APAP regioselectivity in CYP1A2 and CYP2C9. APAP regioselectivity seems to be related to the selectivity for one binding conformation over another binding conformation as dictated by the size and shape of the active site. Additionally, unlike docking and molecular dynamics (MD), our free energy calculations successfully reproduced a unique APAP pose in CYP3A4 that had been reported experimentally, suggesting this approach is well suited to find the realistic binding pose and the lowest-energy starting structure for studying the chemical reaction step in the future.

## Introduction

Enzymes catalyze thousands of chemical reactions. Some enzyme catalysis exhibits interesting product regioselectivity or chemoselectivity. The origin of the regioselectivity has always interested chemists, with the last decades seeing many studies conducted to interpret this characteristic in enzyme catalysis [1] [6]. Computational efforts have also contributed to the understanding of product regioselectivity in chemical reactions and enzyme catalysis [4],[5],[7],[8]. Most computational work has used quantum mechanics (QM) or hybrid quantum mechanics/molecular mechanics (QM/MM) methods to compute and compare the reaction barriers that lead to different products [5],[7]–[9]. Although comparing energy barriers can well explain the regioselectivity for non-enzymatic reactions, chemical energy barriers alone may not be sufficient to elucidate the regioselectivity in enzyme catalysis. Enzyme catalysis is presumed to consist of four steps: (1) ligand diffusing to the enzyme, (2) ligand binding to the enzyme active site, (3) enzyme catalyzing the chemical reaction, and (4) product leaving the enzyme. The first three steps play important roles in deciding the substrate specificity [6],[10]–[13], while both steps (2) and (3) greatly decide the product regioselectivity [14] [15]. In fact, according to the Curtin-Hammett principle [16],[17], a complete understanding of selective product formation should include knowledge from both the chemical reaction step and the equilibrium distribution during substrate binding. Additionally, these two determining factors may or may not play equal roles in product regioselectivity. As a good example, the Merz lab investigated both the relative binding free energies and the reaction energy barriers for NphB catalyzed aromatic prenylation [4],[18]. The authors found that although the transition state (TS) barriers from QM/MM calculation favors the production of the minor product, the enzyme preferentially binds the ligand to a binding state that favors the reaction leading to the major product, with a larger (binding) free energy difference than the TS barrier difference, thus resulting in the experimentally observed regioselectivity. Also, the comparison of productive sites and the unproductive sites in that work suggest that the binding event does the first round of screening for product regioselectivity, thus limiting the number of products, prior to the chemical reaction step which further down-selects to specific products. However, such a study requires extensive computations and has not been widely applied to understand the regioselectivity of a single substrate against a family of enzymes, such as the kinase family or cytochrome P450 (CYP) family. In fact, a substrate can exhibit quite different regioselectivity with different members in the same enzyme family, making the prediction of product regioselectivity in such scenarios more complicated and necessary for complete mechanistic understanding.

CYPs play a vital role in xenobiotic metabolism, which is the conversion of non-natural compounds, e.g., drugs, to compounds that can be excreted by living organisms. Understanding xenobiotic metabolic pathways is essential to understanding drug metabolism and has direct impact on drug pharmacokinetics. Drug metabolism can be divided into three stages: 1) oxidase enzymes catalyze the modification of drug molecules, usually introducing polar groups; 2) transferase enzymes, such as glutathione S-transferases, catalyze the conjugation of the modified xenobiotics to polar compounds; and 3) efflux transporters recognize the conjugated compounds after they are further processed and subsequently excrete them out of cells. Unsuccessful metabolism leads to the accumulation of drug compounds, which can be harmful to the human body. CYPs participate in the first stage of drug metabolism, catalyzing the oxidation of drug substances, and are essential for drug bioactivation and detoxification [19]. According to a recent survey, CYPs account for approximately 75% of all drug metabolism [20].

The activity of CYPs relies on a heme cofactor, bracketed between the L-helix and I-helix, which has been recognized as the signature secondary structural feature of all CYPs [21]. A thiolate group from a cysteine residue forms a strong electrostatic interaction to the iron on the proximal side of the heme complex, tethering it to the enzyme. The iron of the heme cofactor possesses different charge, spin and coordination states during the generic catalytic cycle, which has been extensively studied [22]–[24]. The reactive species is believed to be compound I (Cpd I), a high valent iron-oxo radical complex [25],[26].

Drug compounds are frequently metabolized by multiple CYPs, where each CYP can exhibit different regioselectivity. Interestingly, Lonsdale *et. al.* have shown that Cpd I exhibits similar catalytic power across different human CYP isozymes [27]. Therefore, the ‘intrinsic reactivity’ of different reacting sites for a single drug molecule should be similar across different CYPs. Specifically, the active species model, usually composed of the drug, the Cpd I analog (usually iron-porphyrin), and the analog of the cysteine that coordinates the heme iron, is the same for a single drug in a variety of different CYPs. However, in spite of the similar ‘intrinsic reactivity’ for a given drug, CYPs still show different specificity and product regioselectivity for a given drug compound. Although the CYP-drug binding and CYP-transition state (CYP-TS) interactions are known to determine product regioselectivity between CYPs, the similar “intrinsic reactivity” of CYPs suggests that the equilibrium distribution of binding may play a larger role than the CYP-TS interactions. To identify the difference in substrate specificity and product regioselectivity against a set of CYPs is key to predicting the metabolic outcome of drug compounds and could be extremely useful in drug discovery and development. For example, predicting unwanted and toxic metabolites, in conjunction with CYP panel screening, could decidedly guide lead optimization and mitigate risks during clinical trials.

Mulholland, Harvey and coworkers carried out MD simulations along with QM and QM/MM level calculations to investigate the regioselectivity in CYP catalyzed drug metabolism and revealed that the TS barrier plays an important role in determining product regioselectivity [5],[9]. Their approach, especially the QM/MM approach, was able to capture important selectivity effects, such as CYP-TS interactions, that could not be obtained through many other methods that mainly rely on the standalone relative intrinsic reactivity of different sites of the substrates and/or the proximity of different sites to the heme iron. However, their model does not account for the relative binding free energy differences (ΔΔ*G*_binding_) between reactive binding states (that could lead to different metabolites) within the active site, which was proved to be very important in their CYP2C9-diclofenac example [9]. In fact, CYP2D6 and CYP2C9, which were used in their two studies separately, are known to have voluminous active sites, where many degrees of freedom for the substrate might make the free energy difference between different binding modes small enough such that the chemical reaction barrier could play a more significant role in determining regioselectivity. Thus, the role of binding thermodynamics and how the binding pocket size and shape serve as regioselectivity determinants remain an open question.

Additionally, from the chemistry perspective, a drug compound with multiple oxidizable functional groups can lead to multiple metabolites through CYP catalysis. Many predictors have been developed based on the relative intrinsic reactivity of functional groups. However, these predictors usually overestimate the number of metabolites than are observed in nature, e.g., SmartCYP [28] estimates four reacting sites on APAP for CYP2E1 with the toxic metabolite having the worst score. Therefore, the exact mechanisms as to why CYPs usually do not produce as many metabolites as there are oxidizable functional groups in a substrate are unclear.

**Figure 1:**
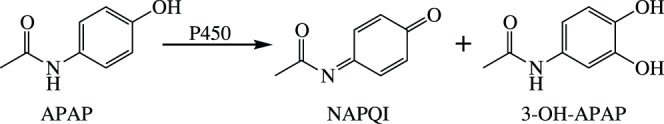
Schematic representation of acetaminophen metabolism by cytochrome P450s.

Calculating relative binding free energies can be computationally intensive because of the large number of degrees of freedom that must be sampled to reach convergence. However, with the increasing abundance of high performance computing (HPC) resources, examining binding free energy landscapes is more feasible for large, flexible binding pockets such as those in CYPs, using well established, albeit computationally intensive, methodologies such as USP. To demonstrate that this approach is viable and useful, we picked acetaminophen (APAP) as our model substrate and applied large-scale USP simulations to examine the free energy binding landscape and its effect on regioselectivity in a set of human CYPs. As a commonly used pain reliever and fever reducer, APAP is well known for its drug-induced hepatotoxicity at high doses [29],[30]. The toxicity is due to the accretion of a highly reactive toxic intermediate, *N*-acetyl-*p*-benzoquinone imine (NAPQI), while the other metabolite, 3-hydroxy-acetaminophen (3-OH-APAP), is not toxic [31]–[33] (Figure 1). APAP is mostly metabolized by CYP1A2, CYP2A6, CYP2C9, CYP2D6, CYP2E1, and CYP3A4. Among them, CYP2E1 is widely accepted as the principal source of NAPQI [34],[35], while CYP1A2 and CYP3A4 are also believed to contribute to NAPQI accumulation [36]. On the other hand, CYP2A6 is proved to principally convert APAP to 3-OH-APAP, and CYP3A4 is also found to actively produce this non-toxic metabolite [35]. APAP has been widely and carefully studied [34],[37]–[41]. The large body of literature provides us a great opportunity to validate our computational results against experimental results. With this in mind, we applied large-scale MD simulations and massive USP simulations to correlate free energy binding landscapes of APAP in several human CYPs, known to preferentially metabolize APAP, to experimentally documented metabolites.

## Computational Methods

In this study we adopted a protocol composed of four parts, including the structure selection and preparation, molecular docking of APAP into CYPs, MD simulations and analysis, and free energy surface (FES) scanning using USP. Five of the six human CYP isozymes that catalyze APAP metabolism, CYP1A2, CYP2A6, CYP2C9, CYP2E1 and CYP3A4, were included in this study. CYP2D6 was not included because no high-resolution (better than 2.6 Å) crystal structures were available when this study was carried out, while the use of low-resolution CYP2D6 structures resulted in large RMSD drift during MD simulations. In order to structure-independently sample the universe of binding schemes, three high-resolution PDB structures (except CYP1A2, see supporting information File S1) were selected for each CYP. Below, we illustrate our protocol using CYP3A4 as an example. More details with regard to other CYPs and a graphic representation of our protocol using CYP3A4 as example can be found in File S1.

### Structure Selection and Preparation

To get a better understanding of APAP in the human metabolic cycle, only human CYP structures were selected. In fact, the significance or relevance of results from animal CYP studies to human tests is still unclear [42]. Three PDBs were selected for CYP3A4, namely 3NXU, 1TQN and 3UA1. Each structure was processed using the protein preparation wizard (including side-chain placement with Prime [43],[44]) in the Schrödinger suite of programs. At this stage, missing residues in the middle of the chain were added, and hydrogen atoms were assigned. An oxygen atom was manually added to the Fe–S axis on the distal side of the heme. Bond length (Fe–O), angles and orders were also manually modified to reproduce these properties reported for the Cpd I species.

### Molecular Docking of APAP into CYP Ternary Complexes

At this stage, APAP was docked into each processed structure by using Glide [45]–[47] in the Schrödinger suite of programs. The top ten ranking poses were saved for each structure. Further screening was made based on similarity and ranking in order to select the final five structures to be used for MD simulations. Significantly different poses were preferred during this screening procedure to capture more diversity of CYP-APAP complex. However, if all poses were already included, and five were not reached, then the next highest ranked pose was selected, until five were reached. Crystallographic water molecules were all kept, unless removed for ligand binding.

### MD Simulations of CYP-APAP Complexes and Analyses

All the kept structures were processed with the LeaP program in the AMBER10 suite of programs [48]. Hydrogen atoms, except those on APAP, were removed before the LeaP process. The enzymes were modeled using AMBER *ff99SB* [49], and the heme cluster was modeled using the force field parameters and charge model derived by Cheatham and coworkers [50]. GAFF [51] was used to model APAP. The atomic charges for APAP were computed following a procedure reported by Kollman and coworkers [52],[53]. For APAP, a QM optimization at the B3LYP/6-31G** level was performed, followed by an electrostatic potential (ESP) calculation at HF/6-31G** level; subsequently, a two-stage restraint ESP (RESP) charge fitting procedure was conducted to derive the point charges. The resulting systems were then solvated in octahedral TIP3P [54] water boxes. Each side of the box is at least 8 Å away from the nearest solute atom. The SHAKE [55] algorithm was applied to constrain all hydrogen involved bonds, and the particle mesh Ewald (PME) [56] method was invoked to treat long-range electrostatics interactions. A weakly harmonic restraint (2 kcal/mol⋅Å^2^) was applied on all enzyme heavy atoms. For each solvated ternary complex, a series of minimizations was performed in order to clear possible close contacts. Subsequently, each system was slowly heated up to 300 K over 150 ps using the Langevin thermostat [57],[58]. The collision frequency of the Langevin thermostat was 5 ps^−1^, and the time step for this stage of canonical ensemble (NVT) simulation was 0.5 fs. Followed was a constant pressure (NPT) simulation of 850 ps with a 1 fs time step to equilibrate the pressure to 1 atm. The weak restraint was gradually removed during the heating and equilibration process. Another 19 ns NPT ensemble simulation with a 2 fs time step was performed followed by a 20 ns NVT simulation to propagate the complexes over more phase space. For each selected PDB ID, five trajectories of the last 39 ns (19 ns NPT and 20 ns NVT) simulations were then merged, resulting in a 195 ns MD trajectory for each PDB ID. Subsequently, the MMTSB [59] program was used to perform the cluster analysis. AMBERTools 12 [60] was used for analysis.

### FES Scan using 2D USP

**Figure 2:**
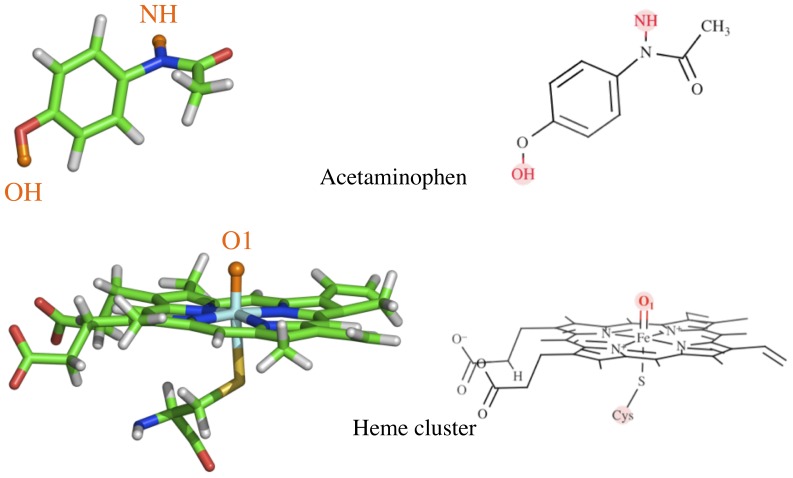
Labeling of important atoms of acetaminophen and the heme cluster.

For each of the five CYPs, a snapshot from the 19 ns NPT simulations was randomly selected as the starting structure for the following 2D USP simulations. Two reaction coordinates (RC) were selected as (1) the distance between the amide hydrogen atom (NH, see Figure 2 for atoms labeling) of APAP and the oxygen atom (O1) coordinating to iron (RC1, *d*_NH-O1_) and (2) the distance between the hydroxyl hydrogen atom (OH) of APAP and the O1 atom (RC2, *d*_OH-O1_). Such a selection allows straightforward identification of conformations representing close proximity of the reactive heme complex to one of the reacting centers of APAP, either the amide nitrogen or the 3-carbon adjacent to the hydroxyl group. Sampling windows were placed along RC1 from 1.6 Å to 11.8 Å and RC2 from 1.5 Å to 12.3 Å, with a 0.6 Å interval for both RCs. Regions that do not make physical sense, e.g., RC1≤1.6 Å while RC2≤1.5 Å, or that were never sampled during MD simulations were excluded. Approximately 280 windows were prepared for each CYP isozyme. An NPT ensemble simulation of 10 ns was carried out at each window in order to investigate the Gibbs FES. A 20 kcal/mol⋅Å^2^ harmonic potential was applied to each RC for all the sampling windows, and data were collected every 0.2 ps during the last 5 ns MD at each window. The weighted histogram analysis method (WHAM) [61]–[63] was applied using Grossfield’s WHAM code [64] to remove the biased potential and reconstruct the free energy profiles. Program R [65] was used to generate the plots for each profile. In order to analyze the CYP-APAP interaction pattern, a short 2ns unbiased MD simulation in the NPT ensemble was carried out for each important binding scheme. All the interactions, including H-bond, APAP-O1 interaction, or possible π interactions are tracked and judged through the simulation. A graphical illustration of this entire procedure described above can be found at Scheme S1 in File S1.

### Computational Requirements

Both MD and USP simulations were performed on the Sierra cluster at Livermore Computing, consisting of 1,856 Intel Xeon EP X5660 nodes (each node has 12 2.8Ghz cores and 24 GB memory) using an InfiniBand QDR interconnect. Each CYP used 145,000 to 180,000 CPU hours to calculate each 2D energy landscape for a total of ∼800,000 CPU hours used to complete this project (see File S1 for more detail about HPC usage. AMBER PMEMD benchmark is shown in Chart S1 in File S1).

## Results and Discussion

### APAP in CYP3A4 Features Two Stable Binding States Connected by a Less Stable Intermediate State and a ‘Flat’ Free Energy Surface

**Figure 3:**
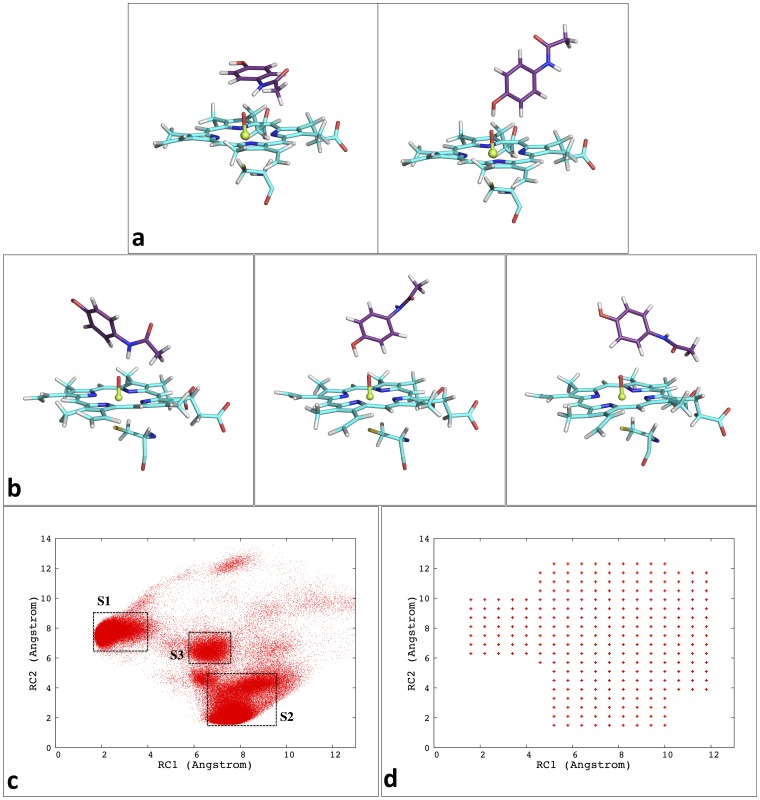
Docking and MD results for CYP3A4-APAP. (a) CYP3A4-APAP docking poses feature close proximity of NH-O1 (left) and OH-O1 (right), carbon atoms of the heme and the iron coordinating cysteine residue are colored in cyan, carbon atoms of APAP are colored in purple, iron is shown as a sphere and color in lime (the same color schemes are also used for all other figures). (b) APAP poses identified from MD simulation trajectories, featuring close proximity of NH-O1 (left) and OH-O1 (middle), and close proximity of the C-3 atom to O1 but with no proximity of either NH-O1 or OH-O1 (right). (c) Distribution of RC1 and RC2 in all 15 MD simulations of CYP3A4-APAP with the S1, S2, and S3 states defined. (d) Distribution of RC1 and RC2 for all USP windows.

Among all the CYP isozymes, CYP3A4 draws the most attention because it can metabolize a wide assortment of substances, including a number of large substrates. Crystallographic studies reveal CYP3A4 has a voluminous active site that can bind large ligands. The large binding pocket also allows CYP3A4 to bind multiple identical ligands or different ligands simultaneously, e.g., two APAPs or APAP and caffeine [66]. Due to the large size of the active site, *in silico* docking of ligands, especially small ligands, into CYP3A4 can be difficult. As expected, APAP exhibits large degrees of freedom in the CYP3A4 active site; docking APAP into CYP3A4 yields more than five different orientations among the ten highest-ranking poses in each of the three PDB structures used to represent CYP3A4. Similarities in binding poses were observed across three structures where at least one pose featured NH closely pointing to O1 and one pose with OH in close proximity to O1 were identified for each PDB (Figure 3a). Differences were also seen across three PDBs, likely due to the slightly different binding pocket structures. This makes our choice of using three PDBs more rational and the final results less dependent on the choice of starting structure. MD simulations starting from the docked poses were subsequently carried out to investigate the dynamics of APAP in CYP3A4. Several amino acid (AA) side chains surround and interact with APAP in the binding sites. R105 and S119 are localized at the propionic acid side, and T309, I369, and A370 stand like a hydrophobic wall on the other side, encircling the APAP pocket, while F304 and L482 appear to form the ceiling for the pocket (Figure S1 in File S1). However, none of these side chains are very close to APAP, resulting in a fairly spacious pocket in which APAP can likely rotate to change orientation. Through cluster analysis, three major APAP binding conformations were identified: (1) NH in close proximity to O1, (2) OH in close proximity to O1, and (3) 3-carbon still close to O1, but neither NH nor OH are in close proximity to O1 (Figure 3b). The distribution of RC1 and RC2 was plotted (Figure 3c). Clearly, three clusters have the largest distributions and are termed as S1, S2 and S3 (the same terminology will also be applied to other CYP-APAP complexes to represent similar conformations). S1 shows a shorter RC1 but longer RC2, resulting in NH being in close proximity to O1. S2 exhibits a shorter RC2 but long RC1, featuring close proximity between OH and O1. S3 likely serves as an intermediate state connecting S1 and S2. Clearly, a larger population density between S2 and S3 (S2<−>S3) is seen compared to the population density between S1<−>S3, possibly suggesting faster interconversion. Although the 15 sets of simulations give a great deal of useful insights into diverse APAP binding states, more information, e.g. free energy, is needed to fully understand CYP3A4-APAP binding and locate correct binding pose or starting points for more computationally expensive QM or QM/MM studies.

**Figure 4:**
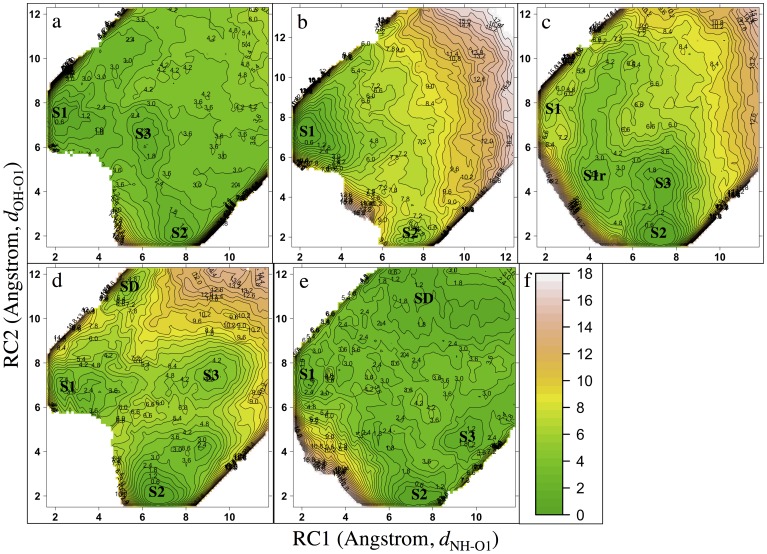
Free energy profiles for CYP-APAP binding. (a) CYP3A4, (b) CYP2E1, (c) CYP2A6, (d) CYP1A2, (e) CYP2C9. Topography energy legend with energy values (kcal/mol) represented by defined color is given in (f). Binding states, S1, S2, S3, S1r and SD are labeled. For CYP2E1, CYP2A6, and CYP1A2, the binding states are clearly defined as energy basins. For CYP3A4 and CYP2C9 where the energy landscape is relatively flat, energy profiles with more color layers are given Figure S4 in Figure S1.

**Figure 5:**
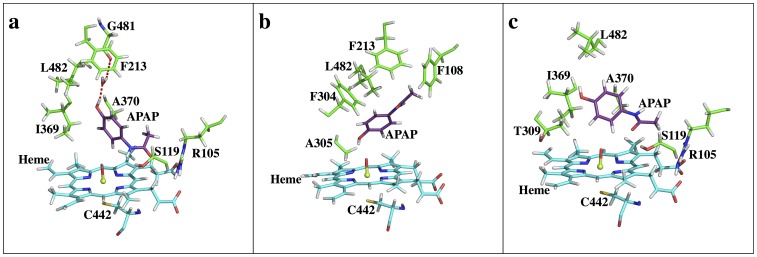
Active sites snapshots of CYP3A4-APAP. (**a**) S1, (**b**) S2, (**c**) S3 binding states. The main chain of enzyme residues (other than iron coordinating cysteine) are omitted for clarity unless it forms a H-bond to APAP or it sits very close to APAP (all other figures are shown in a similar manner).

In order to further investigate various CYP3A4-APAP binding conformations and discover the underlying free energy relationship, USP simulations were carried out. The sampling windows were placed based on the distribution of two RCs, and the range of each RC was selected to be able to cover the APAP movement in the entire binding site (Figure 3d). Processed using WHAM analysis, the resulting free energy profile is plotted in Figure 4a. Clearly, three minima are identified, and their locations are consistent with S1 (RC1 = ∼2 Å RC2 = ∼8 Å), S2 (RC1 = ∼8 Å, RC2 = ∼2 Å) and S3 (RC1 = ∼6 Å, RC2 = ∼7Å) separately, as classified in Figure 3c. S1 has the lowest free energy, and thus, is the most stable binding state. The free energies associated with S2 and S3 are approximately 0.5 and 1.3 kcal/mol higher than S1, respectively. Despite the absolute energy barriers for S2<−>S3 and S1<−>S3 being nearly identical, the interconversion of S2<−>S3 is about 0.7 kcal/mol more favorable than S1<−>S3, confirming the S2<−>S3 interconversion is indeed faster than S1<−>S3 interconversion. The S2 to S3 transition only requires a hydrogen bond (H-bond) to break between the hydroxyl and O1 and a small rotation (see Figure 5). In fact, the ∼0.7 kcal/mol barrier height of S2<−>S3 is consistent with the energy of losing a H-bond (∼1 kcal/mol). Notably, the entire FES is fairly flat and contains several minima-like basins, as would be expected for CYP3A4 which possesses a voluminous active site, exhibits promiscuous ligand binding, and metabolizes approximately half of the currently used drugs [67]. At the S1 state (Figure 5a), the aromatic group lies in a plane vertical to the heme group while the amide nitrogen is in close proximity to O1. Unlike in both docking and MD stages where APAP looks extended, the amide group of APAP is bent away from the phenol plane. This pose nearly reproduces the pose observed in a NMR T_1_ paramagnetic relaxation experiment by the Nelson lab [66], except for the heme propionic acid groups pointing in different directions. In fact, the reported orientation [66] of the propionic acid groups might not necessarily represent the most realistic one because only the distances of the aromatic hydrogen atoms and the methyl group to Fe were measured. The average distance between Fe and hydrogen atoms at 2-carbon atoms is ∼5.2 Å, the average distance between Fe and 3-carbon hydrogen atoms is ∼6.3 Å, and the distance between Fe and three methyl group hydrogen atoms is ∼6.7 Å (averaged over multiple S1-like snapshots). The corresponding values measured in the experimental work are 5.4±0.1 (6.0±0.1, for parallel APAP binding), 5.8±0.1 (6.5±0.1) and 6.4±0.2 (6.4±0.2) Å, respectively [66]. Our result is not only qualitatively but also quantitatively consistent with experimental data. The recovery of this pose only through our free energy simulations further demonstrates the importance of this approach in terms of identifying the realistic ligand binding pose and preparing starting structures for investigating the chemical reaction step. In the absence of free energy simulations, the structures obtained from the MD trajectory are not necessarily representative of the actual binding conformations, making any calculated barrier height less reliable.

In the S1 state, APAP orients itself parallel to the heme plane with the methyl group in close contact to residues R105 and S119. The APAP hydroxyl group forms a H-bond to a water molecule, which donates one hydrogen to form a H-bond with the G481 backbone while the other water hydrogen points to the F213 aromatic ring center (Figure 5a). In addition, a direct interaction between the APAP amide group and O1 is found at the S1 state, making it the most stable binding state. S2 features APAP standing nearly orthogonal to the heme group with the hydroxyl group pointing toward O1. A direct interaction from the APAP hydroxyl group to O1 is observed. The APAP methyl group is surrounded by F108, F213, and F304 (Figure 5b). All three Phe residues belong to the unique Phe cluster [68]. Although the exact function of such a Phe cluster is not yet clear, F108 and F213 appear to stabilize the APAP methyl group via weak CH-π interactions [69]. S3 is directly connected to both S1 and S2 with fairly low barriers (Figure 5c), while the barrier separating S1 and S2 directly is much higher. Therefore, S3 is indeed functioning as an intermediate state. On the other hand, O1 is still close to one of the two possible aromatic hydroxylation centers (carbon atoms ortho to the hydroxyl group), making S3 a possible candidate for 3-hydroxylation. However, APAP is found to neither directly interact with the heme nor to form a H-bond with the protein. Therefore, S3 is less stable than either S1 or S2. At all three states, only a few AA side chains are found in close range to APAP, thus APAP is “free” to move around. Such loose contacts between CYP and APAP may explain why CYP3A4, despite being the most abundant CYP in the human body, is not the major CYP responsible for APAP metabolism. According to the relative free energy differences, the concentration ratio of these three conformations (S1:S2:S3) is close to 6∶2:1. Thus, unless the TS barriers for *N*-oxidation and 3-*C*-hydroxylation are significantly different, both metabolites would be expected. Based on experimental evidence, CYP3A4 does not seem to make significant contributions to NAPQI formation and APAP metabolism [35]. Our calculations are in excellent correlation with this experimental finding (Table 1).

**Table 1:** APAP-CYPs regioselectivity determined by experimental data and calculation.

	Experimental Observation [79]	Prediction suggested by free energyprofile	Prediction after including ‘Intrinsic Reactivity’#
CYP1A2	3-OH-APAP & NAPQI (low activity)	3-OH-APAP	3-OH-APAP slightly favored
CYP2A6	3-OH-APAP (major) & NAPQI (minor)	3-OH-APAP	3-OH-APAP
CYP2C9	3-OH-APAP (low rate) & NAPQI (low activity)	3-OH-APAP	3-OH-APAP & NAPQI comparable
CYP2E1	3-OH-APAP (minor) & NAPQI (major)	NAPQI	NAPQI
CYP3A4	3-OH-APAP & NAPQI	3-OH-APAP & NAPQI comparable*	3-OH-APAP & NAPQI comparable*

# Empirical TS barrier different refers to the estimated energy difference between *N-*oxidation and 3*-C*-hydroxylation from CYP2A6 study.

* Both S2 and S3 could lead to 3-OH-APAP. NAPQI could be slightly favored product.

### APAP in CYP2E1 Exhibits a Remarkably Dominant S1 State

**Figure 6:**
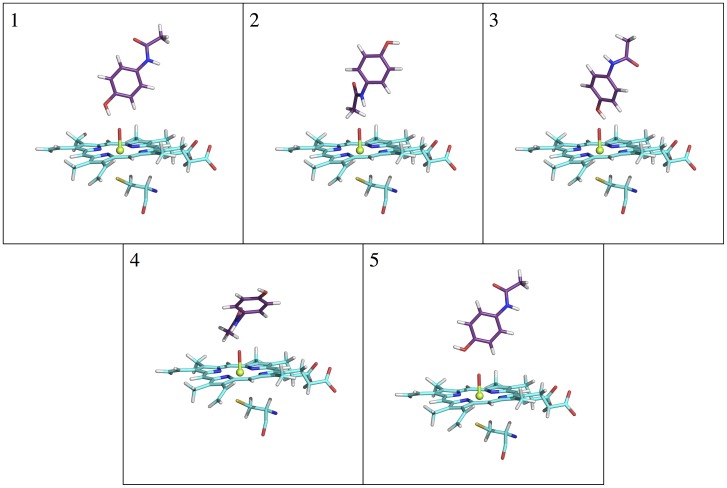
Top 5 (#1–5) scoring binding poses from CYP2E1-APAP docking results. Orientation of the views has been kept the same for easy comparison.

CYP2E1 is far less expressed in the human body than CYP3A4, but is responsible for 30% to 78% of APAP metabolism and is the primary producer of NAPQI [34], the toxic metabolite. Despite numerous studies [34],[41],[70]–[74], the reasons for the high specificity and regioselectivity of CYP2E1-APAP are still not clear. The same workflow as conducted previously for CYP3A4-APAP was carried out for CYP2E1-APAP. Surprisingly, the docking results did not give more S1-like poses (with a short RC1) than S2-like poses (with a short RC2), given that CYP2E1 is well-known for converting APAP to NAPQI. Even more so, the top-scoring pose is S2-like, which should lead to the non-toxic metabolite (Figure 6). SmartCYP was also used to see if it could predict the correct metabolism site; however, the amide group was listed as the lowest-scoring site for metabolism among all four sites predicted. This result further illustrates the need for a complete understanding of the CYP-APAP metabolism mechanism in terms of improving CYP docking and predicting CYP metabolism. Subsequent MD simulations clearly show a higher population of the S1-like conformation than the S2-like conformation. Interestingly, a S3-like cluster is not observed at all, suggesting fast interconversion between different states is probably prohibited in this complex. Many more AA side chains are observed to be within 4 Å of the ligand compared to CYP3A4-APAP (Figure S2 in File S1). Given a much tighter binding pocket is formed for APAP in CYP2E1 than in CYP3A4, we speculate that the compact pocket likely restricts the transition between the two populated binding conformations. The free energy profile (Figure 4b) confirms the dominance of the S1 state, as well as, the absence of the intermediate state S3. The NAPQI precursor (S1: RC1 = ∼2 Å, RC2 = ∼7 Å) is approximately 4.2 kcal/mol more favorable than its non-toxic counterpart (S2: RC1 = ∼7Å, RC2 = ∼2Å). The absence of the intermediate state results in a large free energy penalty (∼7.3 kcal/mol) for the S1<−>S2 transition, making the barrier crossing expectedly ∼1000 times slower than that taking place in CYP3A4. Notably, the free energy increases much more rapidly when APAP moves further away from the iron-heme than is seen in CYP3A4-APAP, implying that APAP has a higher dissociation (off) barrier, and thus a lower dissociation rate (*k*_OFF_) as well as, longer residence time in CYP2E1 than in CYP3A4. The longer residence time of CYP2E1-APAP may allow more significant oxidation activity to form NAPQI than CYP3A4-APAP. The significant preference of the S1 state would lead CYP2E1 to oxidize APAP at the (amide) N site more readily and produce NAPQI, which agrees with experimental findings that CYP2E1 is the principal NAPQI producer.

**Figure 7:**
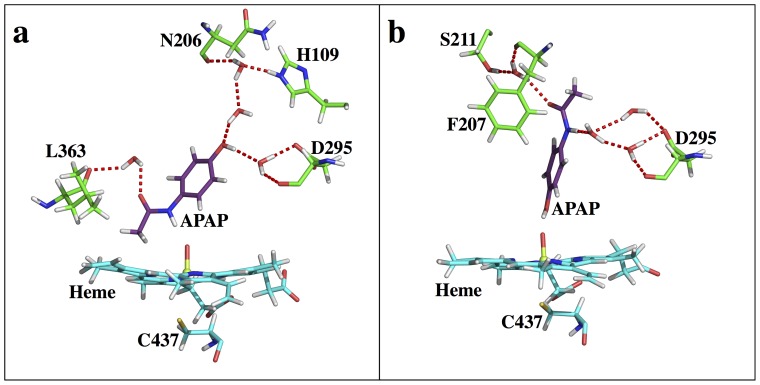
Active site snapshots of CYP2E1-APAP. (a) S1, (b) S2 states.

The CYP2E1 S1 state possesses a pose different than the CYP3A4 S1 pose (Figure 7a), with the methyl group pointing ‘down’ toward the heme while the phenol group heads ‘up’ to the outside of the pocket. As a comparison, S1 in CYP3A4 has the phenol group closer to the heme than the methyl group. Also, at the CYP2E1 S1 state, APAP is more orthogonal relative to the heme plane than that in CYP3A4. In addition to the direct interaction between O1 and the APAP amide group, an interesting water mediated H-bond system is found to stabilize the APAP carbonyl group and L363, and a multiple water involved H-bond system is also identified to build a connection between the APAP hydroxyl group to H109, N206, and D295 (Figure 7a). A294 can also be included in the latter H-bonding pattern (Figure S2 in File S1), though four water molecules separate its backbone carbonyl oxygen from the APAP hydroxyl group. At the CYP2E1 S2 state (Figure 7b), APAP is still more orthogonal to the heme plane than that in CYP3A4. Besides the direct interaction between the APAP hydroxyl group and O1, a two water mediated H-bond system is seen to stabilize the APAP amide group to D295, while the indirect interaction between L363 and APAP is missing. Also, the methyl carbon atom of APAP is only 3.5 to 4 Å away from several heavy atoms of F207, L210 and F298, potentially producing steric repulsion. As a result, S2 is expected to be less stable than S1. An interesting fact is that the APAP aromatic ring nearly takes the same physical space in the CYP2E1 binding pocket in both the S1 and S2 states, even though the remaining part of APAP is in opposite directions (Figure S2 in File S1). The compact pocket of CYP2E1 strongly restrains APAP in a narrow groove, surrounded by I115, F116, F207, L210, D295, F298, A299, T303, L363, V364, and L368. This interesting finding might help explain the failure of docking to predict S1 as the dominant pose, since (1) both the S1 and S2 states occupy a similar space, and (2) no direct H-bond or salt bridge interactions are observed for APAP, except for the single direct interaction between APAP and O1 in both states. Thus, the docking scores are reflective of the strength of the APAP-O1 interaction, where OH-O (S2) is more favored in energy than NH-O. More interestingly, this groove is similar to an experimentally characterized groove where long chain fatty acids can bind into CYP2E1 [75]. The potential of extending itself further out to accommodate long-chain ligands implies this groove might be involved in the ligand-binding pathway. Based on the free energy profile, CYP2E1 is expected to have higher specificity and more specific product regioselectivity than CYP3A4 (Table 1).

### The Curtin-Hammett Principle and APAP Regioselectivity in CYP2A6, CYP1A2 and CYP2C9

**Figure 8:**
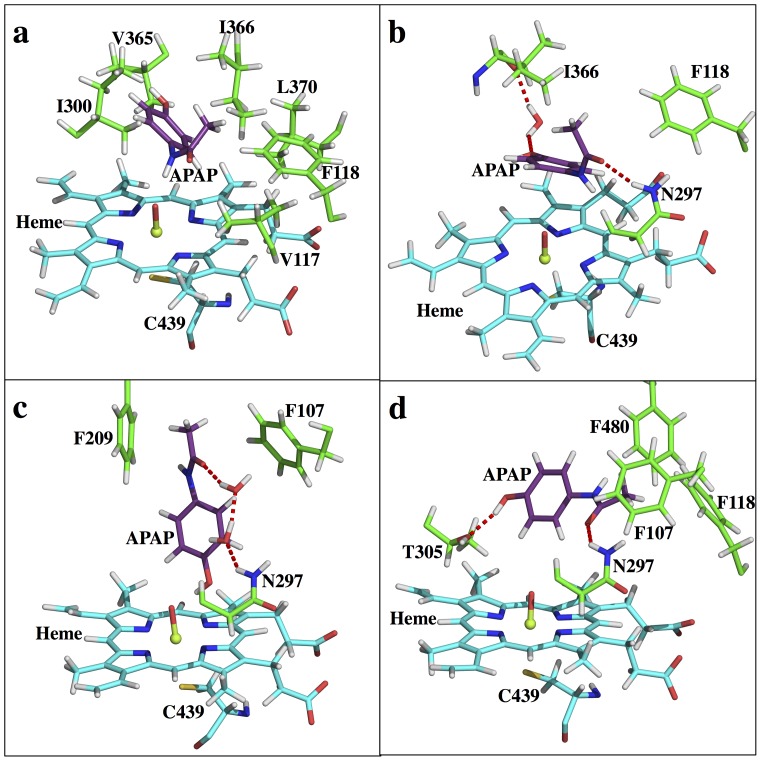
Active site snapshots of CYP2A6-APAP. (a) S1, (b) S1r, (c) S2, (d) S3 states.

CYP2A6 is known to be involved in APAP metabolism, having a less important role than CYP2E1 and preferentially producing the non-toxic 3-OH-APAP metabolite [35]. Although the toxic metabolic NAPQI is also generated by CYP2A6, the product ratio is approximately 1∶3 (NAPQI to 3-OH-APAP) [70]. The FES characterized from USP simulations (Figure 4c) can explain this regioselectivity. S2 (RC1 = ∼7 Å, RC2 = ∼2 Å) clearly has the lowest free energy in the profile; however, the RCs where S1 should be (RC1 = ∼2 Å, RC2 = ∼7 Å) do not represent a stable energy basin. At position RC1 = ∼ 4 Å, RC2 = ∼4 Å, a unique energy basin is identified that possibly functions as a replacement of S1 (S1r) with APAP orientated in a pose nearly ready for the toxic *N-*oxidation. The free energy of S1r is about 2.5 kcal/mol higher than S2. In addition, S3 is identified at RC1 = ∼8 Å, RC2 = ∼4 Å and is about 1.5 kcal/mol less stable than S2, providing an energetically favorable pathway for the S1r<–>S2 interconversion. The protein-ligand, and ligand-solvent interactions can help explain this unique free energy landscape. At the S1 state (Figure 8a) the APAP phenol and methyl groups are strongly pushed by the side chains of V117, F118, I300, V365, I366, and L370 to a position that produces steric repulsion between the two groups. While at the S1r state (Figure 8b), this repulsion is relieved with the methyl group moving away from the phenol group; the distance between the methyl carbon atom and the closest aromatic carbon atom increases from 2.7 (S1) to 3.1 Å (S1r). In addition, at the S1 state the APAP amide group forms a water mediated H-bond network with the N297 backbone oxygen, while at the S1r state the carbonyl oxygen forms a direct H-bond to the N297 side chain. Moreover, a water mediated H-bond between the APAP hydroxyl group and the I366 backbone is observed at the S1r state, but such a H-bond pattern is not seen in S1. Considering the role that I366 played in repulsing the methyl group into a sterically unfavorable position (S1), S1r is expected to be much more stable than S1. At the S2 (Figure 8c) and S3 states (Figure 8d), APAP is further relaxed, resulting in lower free energies. N297 forms a direct H-bond and a water mediated H-bond network with the APAP carbonyl group at the S3 and S2 states, respectively. In addition, a direct H-bond stabilizing the ligand hydroxyl group to O1 and to the T305 side chain is observed in the S2 and S3 states separately. Moreover, at the S2 state, F209 is perfectly placed such that a weak CH-π interaction can form with the APAP methyl group, while at the S3 state such potential CH-π interactions are not found. As a result, S2 is the most stable state for CYP2A6-APAP, and S3 is more energetically favorable than S1r.

**Figure 9:**
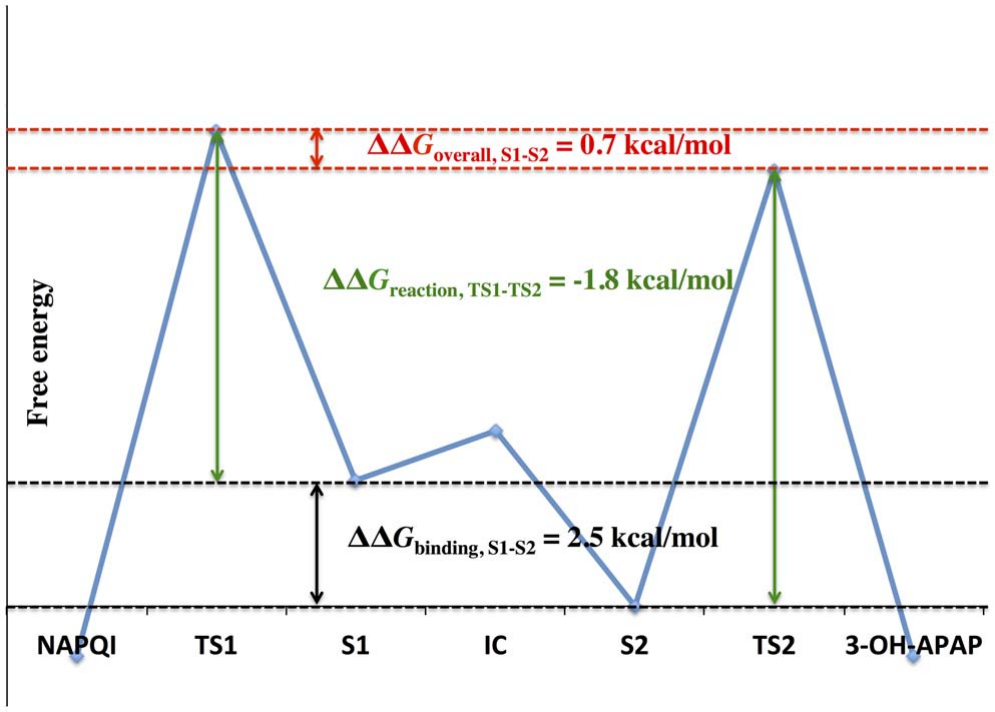
Cartoon representation of the relative free energy for CYP2A6-APAP metabolism according to the Curtin-Hammett principle.

Because the APAP metabolite ratio by CYP2A6 has already been experimentally measured [70] (see also File S1), the free energy profile offers a great opportunity to understand the CYP-APAP metabolism mechanism in terms of the relative free energies of the two reactive conformations at the binding step and in the chemical (reaction) step. Following the Curtin-Hammett principle [17] and using the 1∶3 product ratio [70], the free energy difference in the metabolism cycle, including both the binding and the chemical reaction step, should be about 0.7 kcal/mol favored for the non-toxic metabolism pathway. Thus, based on our calculations, we predict that the reaction barrier for the *N*-oxidation would be about 1.8 kcal/mol lower than the 3-hydroxylation (see Figure 9). Although the energy barrier for the chemical reaction step favors the *N*-oxidation, the 2.5 kcal/mol free energy difference between the two binding equilibriums still makes the aromatic hydroxylation the overall favorable pathway, resulting in a 3-OH-APAP formation preference (Table 1).

According to Hammond’s postulate, the TS is expected to be reactant-like or product-like, depending on whether the chemical reaction is exothermic or endothermic. For reactant-like TSs, the changes in free energy of TS are mainly determined by the changes in free energy of the reactant states and largely unaffected by the changes in free energy of the product state [76]. Since both the *N-*oxidation and *3-C-*hydroxylation are exothermic (see File S1), both TSs are expected to be reactant-like. In fact, in an isotope effect experiment by Nelson and Trager, benzylic hydroxylation, which is similar to *3-C*-hydroxylation, was detected to have a reactant-like TS [77]. Therefore, the changes in free energy from the active species model TS barriers to the CYP-APAP reaction barriers are largely determined by the changes in reactant state from the active species model to the CYP-APAP complex, which is mostly reflected in the binding free energies. As a result, the estimated TS barrier difference for CYP2A6 can be used to interpret the ‘intrinsic reactivity’ of *N-*oxidation to be more favorable than *3-C-*hydroxylation for APAP. Using the “intrinsic reactivity” allows further inference of product regioselectivity for other CYPs, and the CYP1A2-APAP complex is a good example.

**Figure 10:**
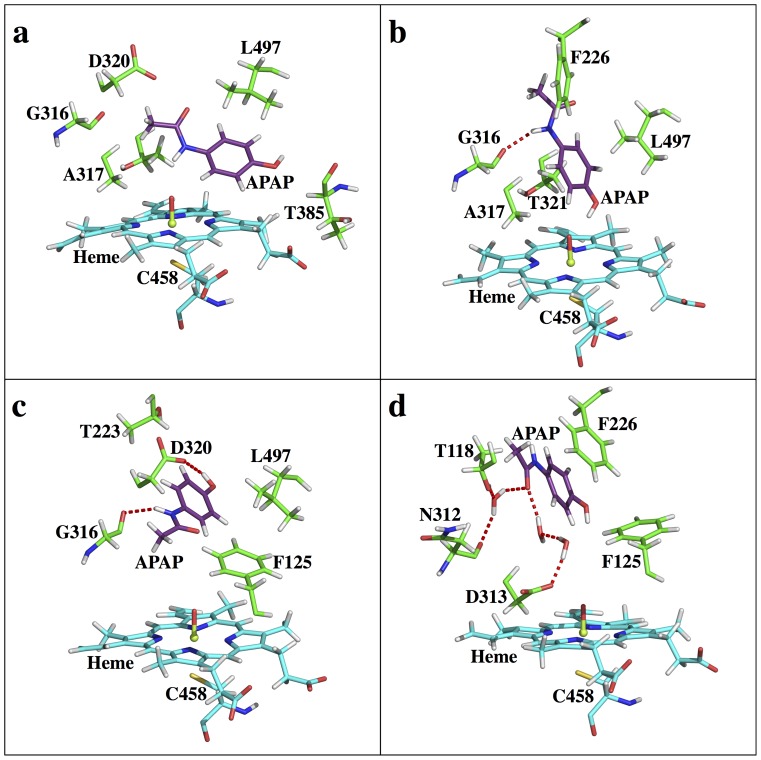
Active site snapshots of CYP1A2-APAP. (a) S1, (b) S2, (c) S3, (d) SD states.

From the currently available experimental results [41],[42],[71],[73] (see File S1), the APAP regioselectivity in CYP1A2 is difficult to interpret. The computationally characterized FES can help explain such regioselectivity. Based on the energy profile (Figure 4d), S2 (RC1 = ∼7 Å, RC2 = ∼2 Å) is more than 2 kcal/mol more favorable than S1 (RC2 = ∼2 Å, RC2 = ∼7 Å), and these two states are connected by an intermediate state, S3 (RC1 = ∼9 Å, RC2 = ∼8 Å), at approximately 4 kcal/mol less stable than S2. Both S3<−>S1 and S3<−>S2 transitions need to overcome ∼5 kcal/mol barriers, while the direct S1<−>S2 conversion is possible, but at an even higher energetic cost (∼6 kcal/mol). Although CYP1A2 S3 and CYP3A4 S3 both function as an intermediate state, the S3 state in CYP1A2 is notably much further away from the heme center than in CYP3A4. The relatively small binding site of CYP1A2 prohibits APAP from free rotation, such that S3 needs to be further away. An interesting observation of this free energy profile is the identification of a distal site (SD, RC1 = ∼5 Å, RC2 = ∼11 Å). This distal site is similar to the distal site reported for human carbonic anhydrase II (HCAII) [78] that serves as an intermediate state along the binding channel; however, the exact function of this CYP1A2-APAP distal site is not clear (see File S1). Overall, since S2 is greater than 2 kcal/mol more favorable than S1, one should expect CYP1A2 to exhibit 3-OH-APAP preference over NAPQI. This conclusion again correlates well with the conclusions in the Rendic review [79] (Table 1), although the real yields to both products could become less differentiated after taking the ‘intrinsic reactivity’ into account. Structurally, CYP1A2 is also known for a compact binding pocket and a narrow entry channel, and the APAP binding locations at all four states fall within a narrow groove (Figure S3 in File S1). At the S1 state (Figure 10a), a direct interaction between the APAP amide group and O1 helps tether the ligand to the heme center. At the S2 state (Figure 10b), direct interactions between the APAP hydroxyl group and O1 and a H-bond between the G316 backbone and the APAP amide group help the ligand gain more stability. At state S3 (Figure 10c), a couple of direct H-bond interactions, namely the APAP hydroxyl to D320 carboxyl and the APAP amide to G316 backbone, help stabilize the S3 state. However, the possible steric repulsion between the hydrophobic side chains of G316, A317 and L497 and the ligand can decrease the stability of this intermediate state, making it energetically less stable than the two reactive states. At the SD state (Figure 10d), a water mediated H-bond system is found between the APAP amide group and T118, N312, and D313. Also, the APAP methyl group is sandwiched by the aromatic rings of F226 and F260, potentially favoring CH-π interactions. The ligand is fully relaxed in this secondary pocket, as no clear steric repulsions can be found (see File S1 for more molecular details). A steep free energy change is again observed when APAP moves away from the heme, suggesting this complex has a small *k*_OFF_ and thus a long residence time that might benefit APAP metabolism. Consistently, CYP1A2 is also a major APAP metabolizer, second only to CYP2E1 [34].

**Figure 11:**
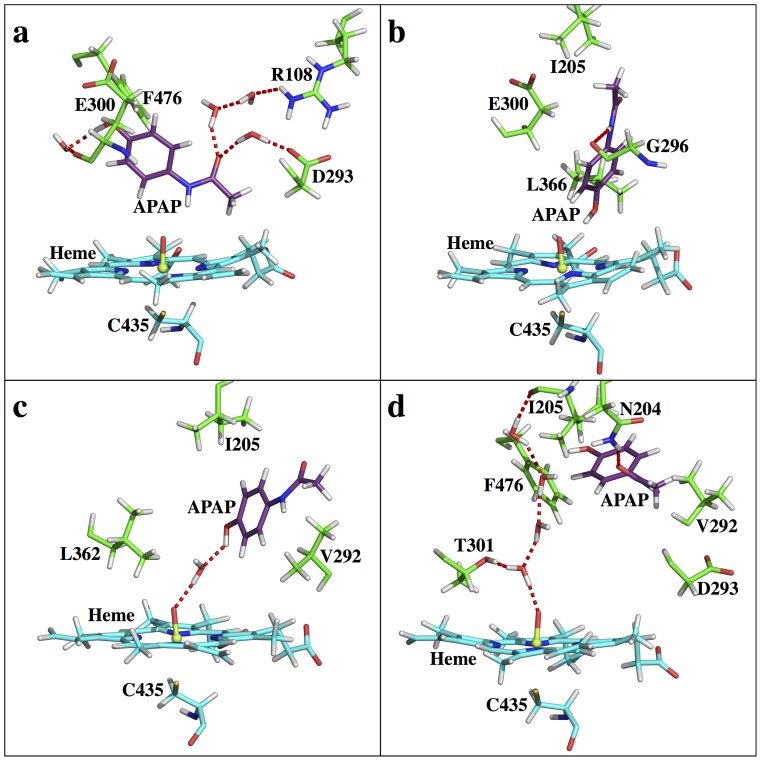
Active site snapshots of CYP2C9-APAP. (a) S1, (b) S2, (c) S3, (d) SD states.

CYP2C9 is another example where our approach can be used to explain APAP metabolite regioselectivity. CYP2C9 is the principal isozyme of the CYP2C subfamily, accounting for ∼18% of the CYP content [80],[81] as expressed in the human liver [80]. Curiously, CYP2C9 does not seem to play an important role in APAP metabolism (see also File S1). As reported in the Rendic review, CYP2C9 is able to metabolize APAP to NAPQI at low activity and to 3-OH-APAP at a low rate [79]. Similar to CYP3A4, which possesses a large binding pocket, the free energy profile of CYP2C9-APAP (Figure 4e) reveals a fairly flat FES, with both the S1 (RC1 = ∼ 7Å, RC2 = ∼ 2Å) and S2 (RC1 = ∼ 2Å, RC2 = ∼ 7Å) states easily identified. A S3 state is also found at RC1 = ∼ 9Å, RC2 = ∼ 4Å. However, unlike in CYP3A4, the CYP2C9 S3 state seems to no longer be an intermediate state for the S1<−>S2 interconversion because another S1<−>S2 conversion route, which does not pass through S3, is energetically more favorable. Instead, the CYP2C9 S3 state likely serves as a local intermediate along the ligand binding pathway. Energetically, S2 is the most stable binding state, meaning CYP2C9 prefers the poses leading toward the non-toxic product. The free energies of S1 and S3 are about 2.0 and 1.6 kcal/mol higher than S2, respectively. Considering the energy barrier of *N-*oxidation is more favorable than aromatic hydroxylation, both metabolites should exhibit activity. Also, the flat FES implies a short residence time for APAP, suggesting that ‘low rate’ and ‘low activity’ [79] might be due to the lack of overall high binding affinities. A distal site (SD) is also observed in CYP2C9 and, according to the energy landscape, is found to be a much bigger area than that seen in CYP1A2. The lowest free energy basin at SD is only <1.0 kcal/mol higher than S2, making it possible to serve as an intermediate along the binding pathway or as a reservoir for ligand binding, similar to CYP1A2 and the function proposed for the low affinity CO_2_ binding site in HCAII [78]. In fact, CYP2C9 is indeed capable of binding multiple ligands simultaneously [82]. Structurally, APAP is surrounded by V113, I205, D293, G296, A297, E300, T301, L362, L366, and F476 in CYP2C9. At the S1 state (Figure 11a), the APAP amide group is tethered to the heme through a direct interaction to O1. Also, water mediated H-bond systems are found to stabilize the APAP hydroxyl group and carbonyl group to the E300 backbone and the D293 carboxyl group, respectively. In addition, a two-water involved H-bond pattern that connects the APAP carbonyl group to the R108 side chain further stabilizes the ligand. At the S2 state (Figure 11b), the APAP hydroxyl group directly interacts with O1, tethering the ligand to the heme cofactor. Also, a direct H-bond between the APAP amide group and the G296 backbone oxygen further stabilizes the ligand. At the S3 state (Figure 11c), neither the hydroxyl nor the amide group of APAP directly interacts to O1, but a water mediated H-bond is found to tether the ligand hydroxyl group to the heme cofactor instead. Also, the APAP amide group and the V292 backbone oxygen form a direct H-bond, although the two sides are approximately 3 Å away. Notably, APAP has many degrees of freedom in the CYP2C9 binding pocket and is surrounded by almost the exact same residues in the S1 and S2 states, making the S1<−>S2 interconversion facile and without requiring APAP to move far from the iron-heme. Therefore, S3 is reaffirmed to be a binding intermediate instead of an intermediate state for the S1<−>S2 interconversion, as in CYP3A4. In fact, the S3 and SD locations (Figure 11d) are close, making this hypothesis more reasonable. At the SD state, the APAP carbonyl oxygen atom forms a H-bond to the N204 side chain. Also, APAP is interestingly positioned such that its aromatic ring possibly forms a weak CH-π interaction with the C_δ_ atom of the I205 side chain, and its hydroxyl group is pointing to the aromatic ring of F476. The ligand might be able to gain affinity through these interactions, resulting in this SD state being suitable as an intermediate state along the binding route. At the SD state, the vacancy of the ligand in the heme pocket is filled out by a structured water system as four water molecules are found to form a H-bond relay that connects the I205 backbone carbonyl oxygen to O1. Overall, CYP2C9-APAP exhibits a FES even flatter than that seen in CYP3A4-APAP (see Figure S4 in File S1). The free energy change upon moving APAP toward the entry of the pocket is less than 3.0 kcal/mol. Therefore, APAP gains less affinity upon binding in CYP2C9 than in CYP3A4, consistently explaining why APAP metabolism by CYP2C9 is concluded as ‘low activity’ and ‘low rate’ [79]. Based on our FES alone, CYP2C9 should exhibit 3-OH-APAP preference over NAPQI (Table 1). This conclusion again shows good correlation with the results in the Rendic review [79].

### Integrating Relative Binding Free Energies and Estimated ‘Intrinsic Reactivities’ to Understanding CYP-APAP Product Regioselectivity

Interestingly, the relative binding free energies alone (the third column in Table 1) seem to show quite good correlation to the APAP product preferences of CYPs as documented in the Rendic review [79]. Two inherent properties may allow for the equilibrium of binding to dominate the product regioselectivity: (1) a given drug should have similar ‘intrinsic reactivity’ for different reacting sites across different CYPs and (2) the relative binding free energies can largely reflect the changes between the difference in ‘intrinsic reactivity’ and the complex TS barrier difference when comparative reactions are exothermic. In fact, according to the calculated ∼2.5 kcal/mol relative binding free energy difference between the S1 and S2 states and the 1∶3 product ratio documented experimentally for CYP2A6, the TS barrier for *N-*oxidation is approximately ∼1.8 kcal/mol lower than *3*-*C*-hydroxylation, while the ‘intrinsic reactivity’ for *N-*oxidation is expected to be a bit more than 1.8 kcal/mol since the enzyme imposed a negative energy difference in binding (S1 to S2) to the change in energy from the ‘intrinsic reactivity’ to the TS barriers. Using ∼1.8 kcal/mol as a reference value (since similar relative binding free energy difference between S1 or S1r and S2 were observed for CYP1A2, CYP2A6 and CYP2C9), the 3-OH-APAP and NAPQI production catalyzed by CYP1A2 and CYP2C9 become much more comparable, with the non-toxic 3-OH-APAP slightly more favorable for CYP1A2 (the fourth column in Table 1). These conclusions show excellent agreement with the documented experimental results [79]. Overall, the CYP-APAP binding seems to play the major role in the different product regioselectivity exhibited across different CYPs.

## Conclusions

MD simulations followed by USP free energy scanning have previously been applied to explain product regioselectivity in a single enzyme [4] but this analysis has not been applied to address the question of product regioselectivity by similar competitive enzymes, as in the case of CYPs. We have applied such an approach to study a single substrate’s (APAP) different product regioselectivity as exhibited in different members of the same enzyme family (CYP) and illustrate how leveraging HPC can lead to new chemical insights via simulation. Human CYPs are responsible for most of drug metabolism [20], thus, the understanding of their regioselectivity is critical to predict metabolite formation, impacting lead optimization and drug clearance. Individual CYPs exhibit different site-of-metabolism selectivities for APAP metabolism. The source of such difference in regioselectivities is examined in this study.

Overall, we have shown that neither docking nor MD simulations can accurately describe the equilibrium distribution of multiple drug binding conformations in CYP-APAP; however, applying free energy simulations and the Curtin-Hammett principle can help explain APAP metabolite regioselectivity. More than sufficient simulation time (see File S1 section II-7 for detailed discussion) was given to each USP window in order to guarantee that the free energy estimates are reliable. The results are consistent with experiments. Our study reveals that each CYP preferentially binds APAP in different poses that are consistent with different sites of metabolism that would lead to different metabolite products.

Several factors play important roles in determining the regioselectivity. First, if the ligand conformation maximally complements the size and shape of the binding pocket, then the substrate affinity and product regioselectivity can possibly be predisposed to specific products even prior to the chemical reaction, such as APAP in CYP2E1. For CYP3A4 and CYP2C9 where the active sites are voluminous, the relatively small ligand APAP is less sterically restrained such that fast interconversion between reactant binding states is allowed, and the ligand has few direct interactions with CYP, consequently decreasing the binding affinity. While for CYP2E1 and CYP1A2 with well-known compact binding pockets, APAP gains more affinity from more interactions with the enzymes than in CYP3A4 or CYP2C9, resulting in stabilized binding states and longer residence time in the active site. Also, having more protein-ligand interactions forces APAP to adopt a highly preferred binding conformation, while transitions between different binding states are likely prohibited. Second, although the proximity of the ligand’s reacting atoms to the heme iron center might not be the major determinant of selectivity in a single CYP, the APAP metabolite regioselectivity across multiple CYPs gives interesting correlations with the distances between the reactive center and the heme iron center. At the CYP2E1 S1 state, the distance between the APAP amide nitrogen and O1 is only 3.2 Å, approximately 0.2 Å shorter than the corresponding distance in CYP1A2 and CYP2C9 and more than 0.7 Å shorter than the corresponding distance in CYP2A6 and CYP3A4. So, not surprisingly, CYP2E1 is the principal contributor to NAPQI accumulation. On the other hand, the distances between the nearest aromatic 3-C atom of APAP and O1 at the S2 state in CYP2A6 is at approximately 3.3 Å, <0.1 Å shorter than that in CYP2C9 and more than 0.2 Å shorter than in CYP1A2, CYP2E1 and CYP3A4. Expectably, CYP2A6 is the proven principal producer of 3-OH-APAP. Third, structural factors might also affect the free energy landscape and, thus, impact the regioselectivity. A representative example is CYP2A6-APAP; the bulky side chain of I366 pushes the APAP methyl group toward the phenol group to result in significant steric repulsion, thus destabilizing the S1 state, while the same I366 backbone interacts with APAP via a water mediated H-bond system to help stabilize the S1r state (see also File S1).

Despite the large number of existing studies on the CYP-APAP metabolism [66],[70]–[74],[83]–[85], new and interesting insights are revealed through our free energy approach. First, we identified at least two binding equilibriums in all five CYP-APAP complexes in this study, including both reactive conformations and a few interesting states such as S1r in CYP2A6 and SD in CYP1A2 and CYP2C9. More importantly, the poses we identified would lead to specific metabolic products that successfully reproduce those metabolites reported experimentally [66] for each CYP. Second, applying the Curtin-Hammett principle helps predict the ‘intrinsic reactivity’ for both reactions. Although calculations at a QM or QM/MM level are required to accurately describe the chemical reactions, our projected regioselectivity based on FES alone shows good correlation with experimental results, and taking the ‘intrinsic reactivity’ into consideration further validates our prediction against experimental results [79]. Third, our results demonstrate how the shape of the binding site and the protein-ligand interactions remarkably affect the ligand *k*_OFF_ and the ligand residence time, which could either increase or decrease the affinity. The fact that CYP1A2 and CYP2E1 have lower *k*_OFF_s and play a more principal role in APAP metabolism, while CYP2C9 and CYP3A4 have higher *k*_OFF_s and play less of a role, again matches experimental observations [35]. Finally, the binding equilibrium between the S1 and S2 states in small-active-site CYPs, such as CYP1A2 and CYP2E1, plays a more significant role in determining APAP regioselectivity; for CYP2C9 and CYP3A4 which possess large active sites, the relative binding free energies between the two reactive states are close such that the chemical reaction step possibly plays a more important role in determining the regioselectivity. In those cases where the chemical reaction step is determinate, the binding poses identified at the basins on the free energy profile can be further used as more reliable starting points for any QM or QM/MM calculations to characterize the reaction mechanism and energies. Other studies have shown success using this strategy [18],[86], and thus the QM and QM/MM calculations starting from the low basin conformations are underway in our lab.

The overall approach utilized is this study is general, and the use of APAP metabolism by CYPs as a case study allows for validation of the approach with a plethora of experimental results. With the ever-improving power of HPC, we expect this approach to become more affordable (see File S1) and to be applied to other protein-ligand complexes, especially single substrates that are catalyzed by a family of enzymes. Moreover, this approach can be also extended to understand drug-drug interaction, such as negative cooperativity of APAP in CYP3A4 when more than one APAP is present, which is also underway in our lab [66]. The growing understanding of substrate binding and product regioselectivity benefits multiple aspects in drug discovery, e.g., improving docking algorithms, attaining more accurate QM or QM/MM results, and predicting more reliable ADME (absorption, distribution, metabolism and excretion) outcomes. Most importantly, this study shows that although TS energy barriers can affect product regioselectivity during the chemical reactions step [5],[9],[18], the binding events prior to the chemical reaction step can significantly direct product regioselectivity and, thus, limit the number of metabolites or products.

## Supporting Information

File S1 **Supplementary schemes, figures, charts and high performance computing usage information are provided in File S1.** This material is available free of charge via the Internet at http://www.plosone.org.
(DOCX)
