# Supplementary material for: Understanding a Substrate’s Product Regioselectivity in a Family of Enzymes: A Case Study of Acetaminophen Binding in Cytochrome P450s
Source: PLoS One. 2014 Feb 3;9(2):e87058. doi: 10.1371/journal.pone.0087058 (PMC3911926; doi:10.1371/journal.pone.0087058)
Supplement: File S1 — Supplementary schemes, figures, charts and high performance computing usage information are provided in File S1. This material is available free of charge via the Internet at http://www.plosone.org. (DOCX) [file pone.0087058.s001.docx]

File S1 (Supporting Information)

for

Understanding a Substrate’s Product Regioselectivity in a Family of Enzymes: A case study of Acetaminophen Binding in Cytochrome P450s

Yue Yang, Sergio E. Wong, Felice C. Lightstone

Biosciences and Biotechnology Division, Lawrence Livermore National Laboratory, 7000 East Ave., Livermore, CA, 94550, USA

**Index**

1. Abbreviations
2. Additional Computational Details and Discussions
3. Supplementary Figures
4. References

**I. Abbreviations**

APAP: Acetaminophen

MD: Molecular dynamics

USP: Umbrella sampling

QM: Quantum mechanics

HPC: High performance computing

LC: Livermore computing center

CYP: Cytochrome P450

**II. Additional Computational Details and Discussions**

**Computational detail.** The PDB code, resolution, and complexed ligands for each selected PDB structure are listed below:

For CYP1A2, PDB ID 2HI4[[1](#_ENREF_1)] (1.95 Å, complexed with α-naphthoflavone) was selected. This is the only high-resolution crystallographic structure available from the RCSB protein data bank.

For CYP2A6, PDB ID 2FDV[[2](#_ENREF_2)] (1.65 Å, complexed with *N*-methyl(5-(pyridin-3-yl)furan-2-yl))methanamine), 2FDU[[2](#_ENREF_2)] (1.85 Å, complexed with *N,N*-dimethyl(5-(pyridin-3-yl)furan-2-yl))methanamine), and 1Z10[[3](#_ENREF_3)] (1.90 Å, complexed with coumarin) were selected.

For CYP2C9, PDB ID 1R9O[[4](#_ENREF_4)] (2.00 Å, complexed with flurbinprofen), 1OG5[[5](#_ENREF_5)] (2.55 Å, complexed with *S*-warfarin) and 1OG2[[5](#_ENREF_5)] (2.60 Å, complexed with *S*-warfarin) were selected.

For CYP2E1, PDB ID 3E6I[[6](#_ENREF_6)] (2.20 Å, complexed with indazole), 3T3Z[[7](#_ENREF_7)] (2.35 Å, complexed with pilocarpine) and 3E4E[[6](#_ENREF_6)] (2.60 Å, complexed with *4*-methylpyrazole) were selected.

For CYP3A4, PDB ID 3NXU[[8](#_ENREF_8)] (2.00 Å, complexed with ritonavir), 1TQN[[9](#_ENREF_9)] (2.05 Å, apo structure) and 3UA1[[10](#_ENREF_10)] (2.14 Å, complexed with bromoergocryptine) were selected.

Although CYP2D6 has also been reported for APAP metabolism activity, it was not included in this study because there were no PDB structures of human CYP2D6 with good resolution (< 2.60 Å) available from the RCSB protein data bank at the time of this study. (The structure with PDB code 3TBG was deposited later, in August 2012).

**Error Estimation of the PMFs.** The statistical uncertainty of each PMF was estimated using a Monte Carlo bootstrap analysis. For all five PMFs, the statistical error is less than 0.5 kcal/mol for most of the regions, while for all regions the error is less than 1.0 kcal/mol.

**CYP2A6**. Although the product ratios for both CYP2A6 and CYP2E1 were measured in the same work[[11](#_ENREF_11)], the ratio for CYP2E1 is strongly impacted by the presence of cytochrome *b_5_,* while the ratio for CYP2A6 metabolism is almost unperturbed[[11](#_ENREF_11)]. Therefore, we believe CYP2A6 is more appropriate than CYP2E1 to be used to understand the free energy landscape for APAP metabolism.

**CYP1A2. (1) Experimental results for CYP1A2 catalyzed APAP metabolism are confusing.** Monostory and coworkers reported that the inhibition of CYP1A2 and CYP3A4 in vitro failed to reduce NAPQI production[[12](#_ENREF_12)]. On the other hand, Zaher et al. found CYP1A2 and CYP2E1 double-null mice were protected against APAP caused toxicity[[13](#_ENREF_13)]. Snawder et al. concluded that CYP1A2 activity increased at high dose of APAP[[14](#_ENREF_14)], and in contrast, Wright and coworkers claimed that CYP1A2 inducers did not increase the NAPQI production after therapeutic dosage of APAP among human volunteers[[15](#_ENREF_15)]. These claims look confusing and a bit contradictory. As a result, the CYP1A2-APAP binding preference and product selectivity remain unclear.

**(b)** **The CYP1A2 S3 state plays a different role than the intermediate state between two reactive binding states.** S3 is distant from the heme center, illustrating flexible rotation of APAP and fast interconversion between S1 and S2 are likely prohibited in this enzyme, as well as in CYP2E1. The locations of the SD and S3 states are close, implying such a site indeed can function as an intermediate state during the ligand binding process or ligand orientation conversion. The fact that SD and S1 are rarely overlapping makes it possible to hypothesize CYP1A2 could simultaneously bind two APAPs, or APAP along with another small ligand, such as nicotine. Although this assumption that multiple APAPs could bind simultaneously may help explain the observation that CYP1A2 activity (not limited to NAPQI production) increases at higher doses of APAP, the removal pathway of the metabolite seems blocked upon ligand binding at the SD location.

**(c) Molecular details of different APAP binding states in CYP1A2.** At the S1 state (Figure 7a), the APAP phenol group is flanked by several hydrophobic residues, including L382, T385, I386, L497, and T498, while the methyl group is surrounded by F226, A317, and D320. At the S2 state (Figure 8b), side chains of A317, T321, L382, I386, L497, and T498 encircle the ligand phenol group, and T223, F226, and V227 are within close range to the methyl group. At state S3 (Figure 8c) or SD (Figure 8d), the ligand is fully surrounded by enzyme side chains, including F125, T223, F226, V227, G316, A317, D320, L497, and T498.

**CYP2C9.** **(a) APAP metabolism activity in CYP2C9.** Patten et al. claimed that no APAP activation by CYP2C9 was observed in their study across a set of human CYPs[[16](#_ENREF_16)]. However, in the same study CYP2D6 was found to have no activity[[16](#_ENREF_16)], whereas a later study confirmed that CYP2D6 is involved in APAP bioactivation[[17](#_ENREF_17)]. Similarly, a recent review listed CYP2C9 as able to metabolize APAP to NAPQI at low activity and 3-OH-APAP at a low rate[[18](#_ENREF_18)].

**(b) Docking and MD results are not sufficient to determine APAP binding preference in CYP2C9.** CYP2C9 is also known for having a large active site and APAP is expected to have many degrees of freedom and show multiple binding conformations. Therefore, with only docking results and MD simulations, the binding selectivity of the CYP2C9-APAP complex is inconclusive because conformations that lead to both NAPQI and 3-OH-APAP are observed.

**(c) Molecular details of CYP2C9-APAP binding states.** At the S1 state (Figure 9a), APAP is in close contact with the side chains of V113, I205, D293, G296, A297, E300, T301, L362, L366, and F476. At the S2 state (Figure 9b), L366 and F476 move 4 Å away from the ligand, possibly resulting in less steric repulsions. At the S3 state (Figure 9c), APAP is surrounded by R108, V113, I205, V237, M240, V292, D293, G296, A297, L366, and F476. While at the SD state, APAP is further away from the heme binding pocket and surrounded by F100, L102, R108, V113, N204, I205, L208, V292, D293, and F476.

**Both NAPQI and 3-OH-APAP formation are exothermic.** QM calculations at CAM-B3LYP/LACVP level were performed to investigate the potential energy difference between the reacting state and product state for both *N-*oxidation and *3-C-*hydroxylation. The QM model consists of the heme cluster analog and acetaminophen, as described in a recent review by the Han group[[19](#_ENREF_19)], with only CH_3_S^-^ instead of HS^-^ was used to represent the iron coordinating cysteine residue. The reaction field calculations were performed at both low-spin doublet and the high-spin quartet state, with the so-called IEFPCM model (polarized continuum model using the integral equation formalism variant) applied. The results show that at both spin states, the *N-*oxidation product state is energetically more stable than its reactant state for more than 20 kcal/mol, while the product state for *3-C-*hydroxylation is more than 40 kcal/mol lower than its reactant state. Therefore, it is rational to conclude both reactions are exothermic. More QM and QM/MM calculations focused on the chemical reaction step are ongoing in our lab and will be reported in the near future.

**Several structural factors can impact CYP-APAP regioselectivity.** Although the structurally conserved CYPs show little sequence conservation, comparison of the protein-ligand interactions across different CYP-APAP complexes still draws our interest and, in fact, provides some valuable insights. First, all CYPs listed in this study invoke interactions with aromatic side chains, mostly phenylalanine, e.g., F108, F213, and F304 in CYP3A4; F116, F207, and F298 in CYP2E1; F107, F111, F118, and F209 in CYP2A6; F125 and F226 in CYP1A2; and F100 and F476 in CYP2C9. In addition, a unique Phe-cluster distantly composed of 7 Phe residues above the heme pocket has been identified for CYP3A4[[20](#_ENREF_20)], and a similar but much smaller cluster containing three Phe residues (F100, F114, and F476) is observed for CYP2C9[[21](#_ENREF_21)]. Although the exact function of the Phe-cluster and the Phe residues close to the APAP binding pocket is not conclusive without a specific study, an educated and aggressive assumption is that those aromatic side chains can steer APAP orientation during the course of binding to encourage the best fit to the pocket. Second, Leu and Ile residues located near the K helix, one of a limited number of sequence conserved regions, are found to play an important role in APAP binding. Examples include I369 in CYP3A4, L363 and L368 in CYP2E1, I366 and L370 in CYP2A6, L382 and I386 in CYP1A2, and L362 and L366 in CYP2C9. APAP can form hydrophobic interactions to the side chains of these residues, or H-bond to the backbones, or weak CH-π interactions, resulting in either favorable or undesirable protein-ligand interactions. A good example is the steric repulsion and the indirect H-bond interaction between the I366 and APAP in CYP2A6 at the S1 and S1r binding state, respectively. Third, charged or polar AA residues, usually from the highly rigid I helix, can help stabilize CYP-APAP complexes. Such examples include D295 and T303 in CYP2E1, N297 in CYP2A6, D320 in CYP1A2, and N204 and D293 in CYP2C9. APAP is more likely to form a direct H-bond with these types of side chains than residues that are not polar or charged. Finally, water molecules (especially crystallographic) play an important role in stabilizing the CYP-APAP binding complex via H-bond interactions. In CYP2E1, water-mediated H-bond patterns are found to tether APAP to the enzyme AAs, helping to gain high affinities for the complex. Similarly, water-mediated H-bond systems are also found in other CYP-APAP complexes. In fact, water in the active site of CYP-APAP complexes may also play a catalytic role in *N*-oxidation to mediate hydrogen hopping, as reported in similar reactions[[22](#_ENREF_22)]. Also, in the CYP2C9 SD state, a four-water-mediated H-bond chain is found to fill up the space left by the absence of APAP, connecting the heme complex to the enzyme environment. Systematically analyzing these protein-ligand interactions also helps explain the different binding preferences and the product regioselectivities for the set of CYPs.

**Additional discussion about large-scale USP simulations adopted in this study.** This research reveals that reliable estimation of relative free energy difference between different binding states and thorough analysis of substrate and CYP interactions are crucial to the understanding of metabolite selectivity and, in the case of APAP, toxic drug metabolite predictions. The five 2-D USP free energy scans in this study consumed about 800,000 CPU hours (see below for details). However, in fact, both the overlap between different sampling windows and the equilibration at each window could be well restored with fewer windows (~1/3) and shorter simulation lengths (1-2 ns). In a recent article describing standard computing binding free energies using PMF-based approaches, Gumbart et. al. reported their success with about 1 ns simulation for each window[[23](#_ENREF_23)]. Therefore, the total number of windows and the sampling time at each window could be significantly reduced to make this approach much more computational efficient. An educated estimation is that the CPU hours associated with these five 2-D USP simulations could be decreased to about 150,000. In addition, because a large number of sampling windows could be simulated simultaneously, this approach is highly ‘parallel’. Thus, this robust free energy approach is highly suitable for high performance computing (HPC) and could play more important roles in future drug discovery predictions.

**Details about computational requirement and HPC usage in this study.** All calculations, including MD simulations, USP simulations, and QM calculations, were performed on Sierra, one of the High Performance Computing (HPC) clusters at Livermore Computing (LC). Sierra is an Intel Xeon EP X5660 architecture based cluster composed of 1,944 nodes and a total of 23,328 cores, recording 20,132 TFLOP/s. PMEMD, a faster MD engine integrated in the AMBER suite of programs, has been utilized for most of the MD and USP simulations. PMEMD exhibits strong scaling to 30 nodes (360 cores, see Chart S1, tests on more nodes have not been made) on Sierra. For USP simulations, most of the windows can be simulated simultaneously by bundling many individual simulations together. For example, in the CYP3A4 case, ~280 USP windows of simulations were bundled together with each window of simulation assigned to 4 nodes (48 cores), resulting in a single job requesting ~1120 nodes. All of ~280 simulations finished within 12 hours. As a result, a total of approximately 160,000 CPU hours were utilized for CYP3A4 simulations. Depending on the number of USP windows required by each APAP-CYP complex, the HPC usages varied from 145,000 to 180,000 for the other four CYPs. These simulations summed up to a total of 800,000 CPU hours of HPC usage. However, as we mentioned in the main text, the simulation time of each USP window can be significantly decreased, resulting in less expensive HPC usage (e.g., a total of 30,000-40,000 CPU hours is estimated for the CYP3A4 case). At this expense level, routine free energy simulations can be expected on HPC.

**II. Supplementary Schemes, Figures and Charts**


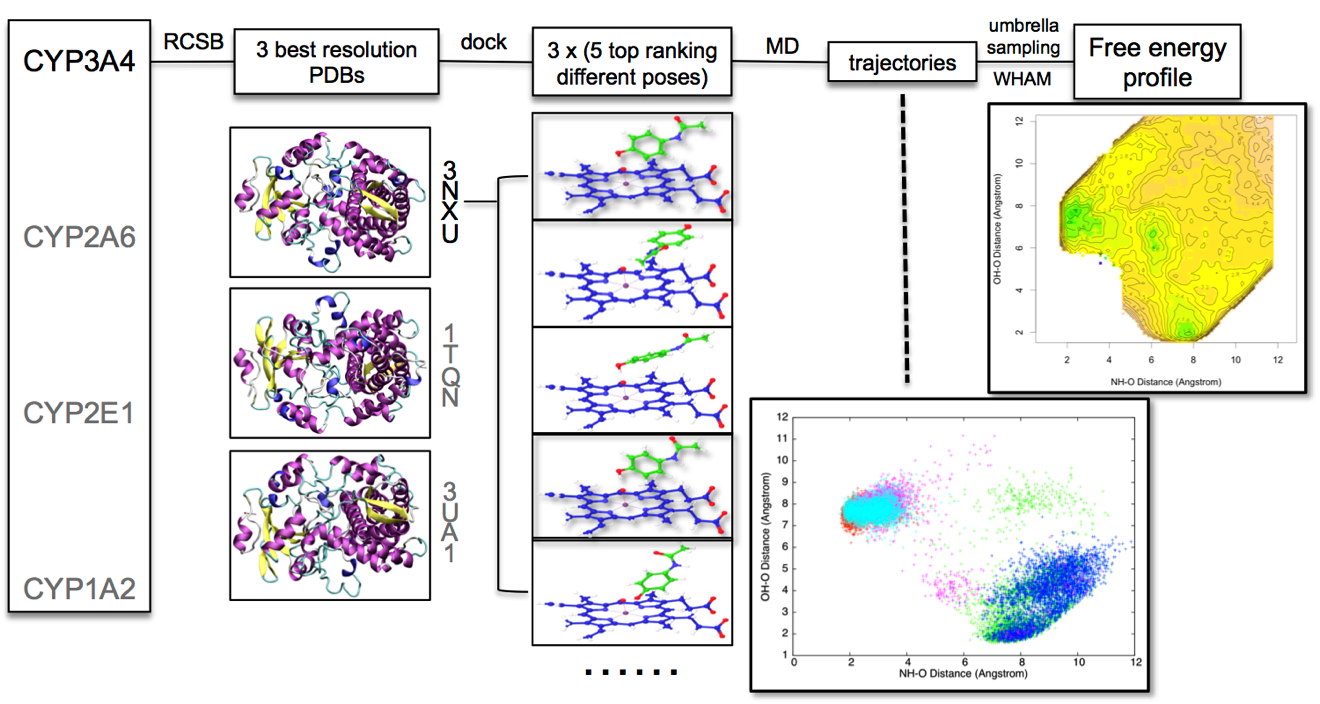


Scheme S1. Graphic illustration of the procedure of our study on APAP-CYP. Small pictures within the scheme are just examples of PDB structures, docking poses, RC distribution map (from MD) and free energy profile.


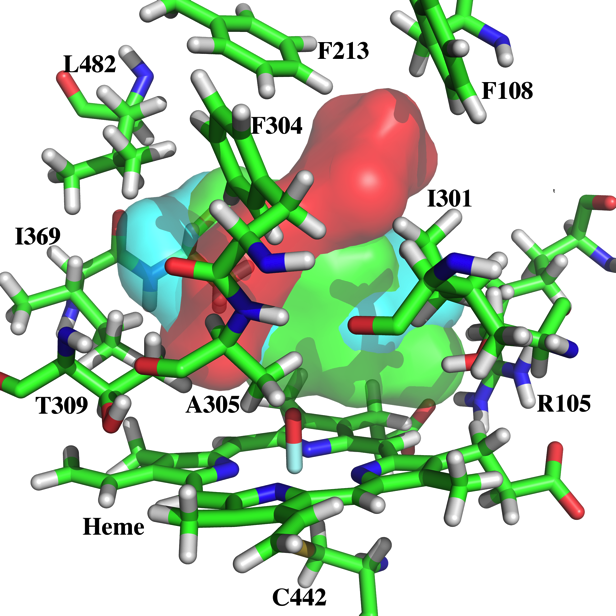


**Figure S1**. APAP (in surface representation) at S1 (green), S2 (red), and S3 (cyan) states in the binding pocket of CYP3A4. (Please note the color scheme of APAP, the heme and iron coordinating cysteine is different to that used in the main text.)

a.
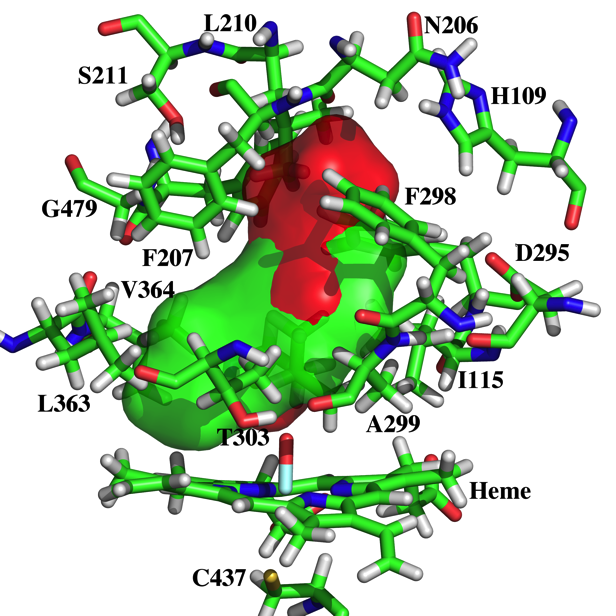
b.
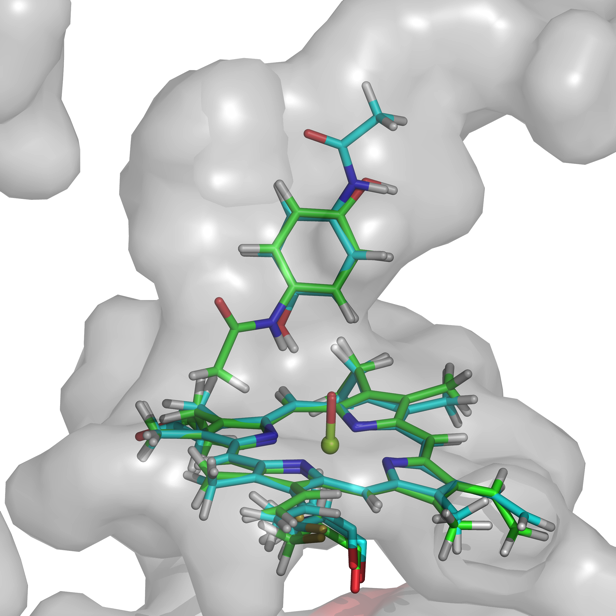


c.
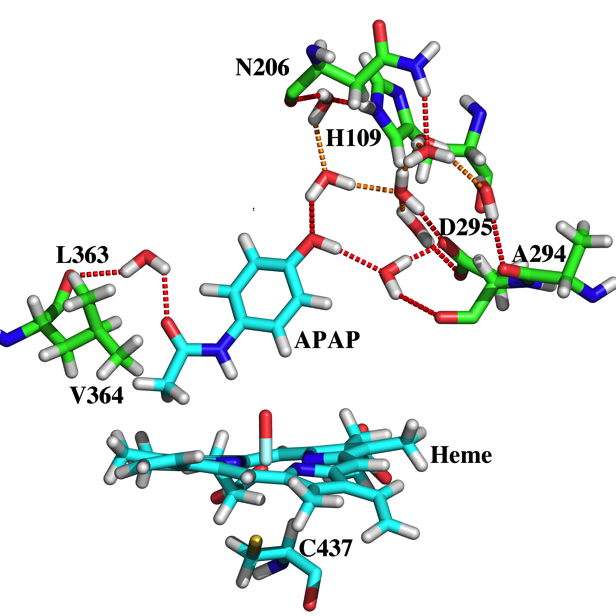


**Figure S2.** (a) APAP (in surface representation) at the S1 (green) and S2 (red) state in the binding pocket of CYP2E1. (b) Graphical illustration of the overlap of the APAP phenol ring at the S1 (carbon atoms in green) and S2 (carbon atoms in cyan) states in the APAP-CYP2E1 complex, with the binding pocket in surface representation. (c) Water mediated H-bond patterns at the APAP-CYP2E1 S1 state.


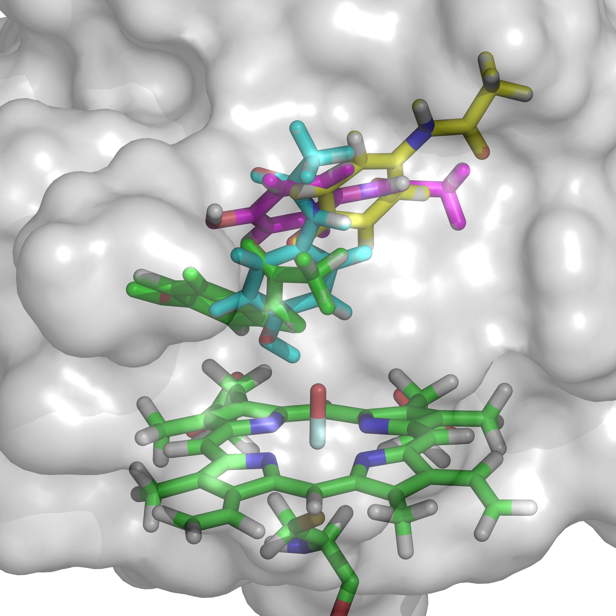


**Figure S3.** APAP at S1 (carbon atoms in green), S2 (carbon atoms in cyan), S3 (carbon atoms in pink) and S4 (carbon atoms in yellow) in the binding pocket (in surface representation) of APAP-CYP1A2 complex.


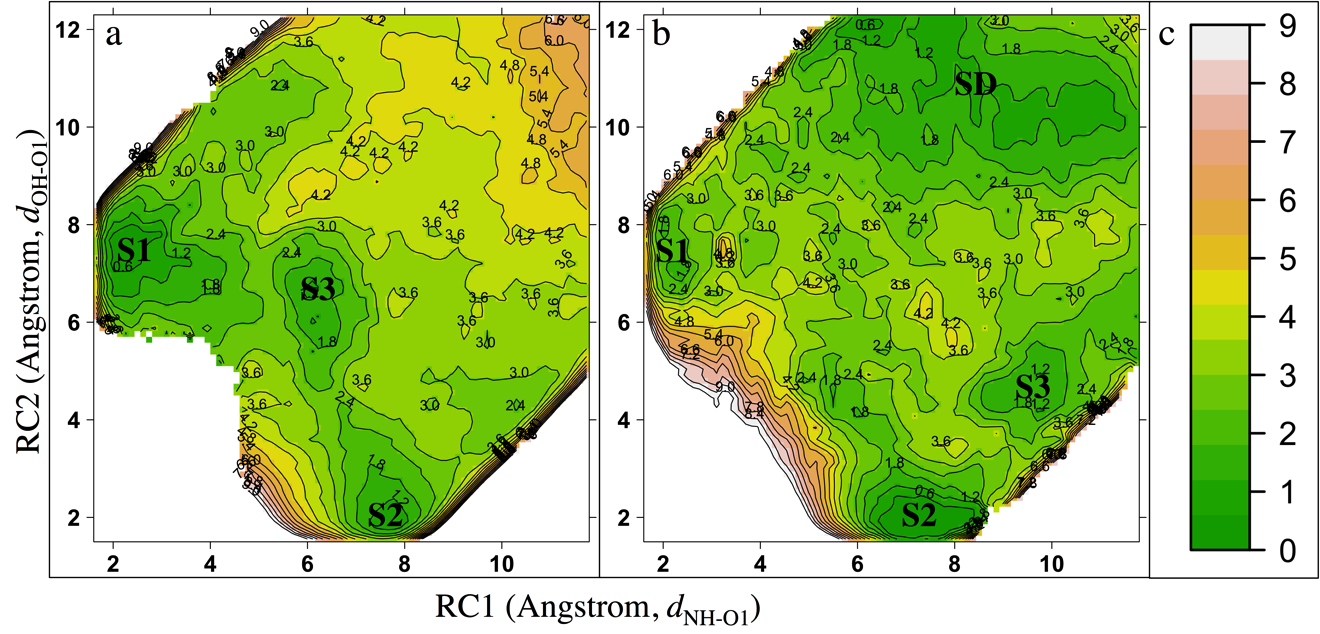


**Figure S4.** Free energy profiles for APAP binding in (a) CYP3A4 and (b) CYP2C9, (c) defines the color scheme (note it is different to the color scheme in Figure 2) and the unit is kcal/mol.


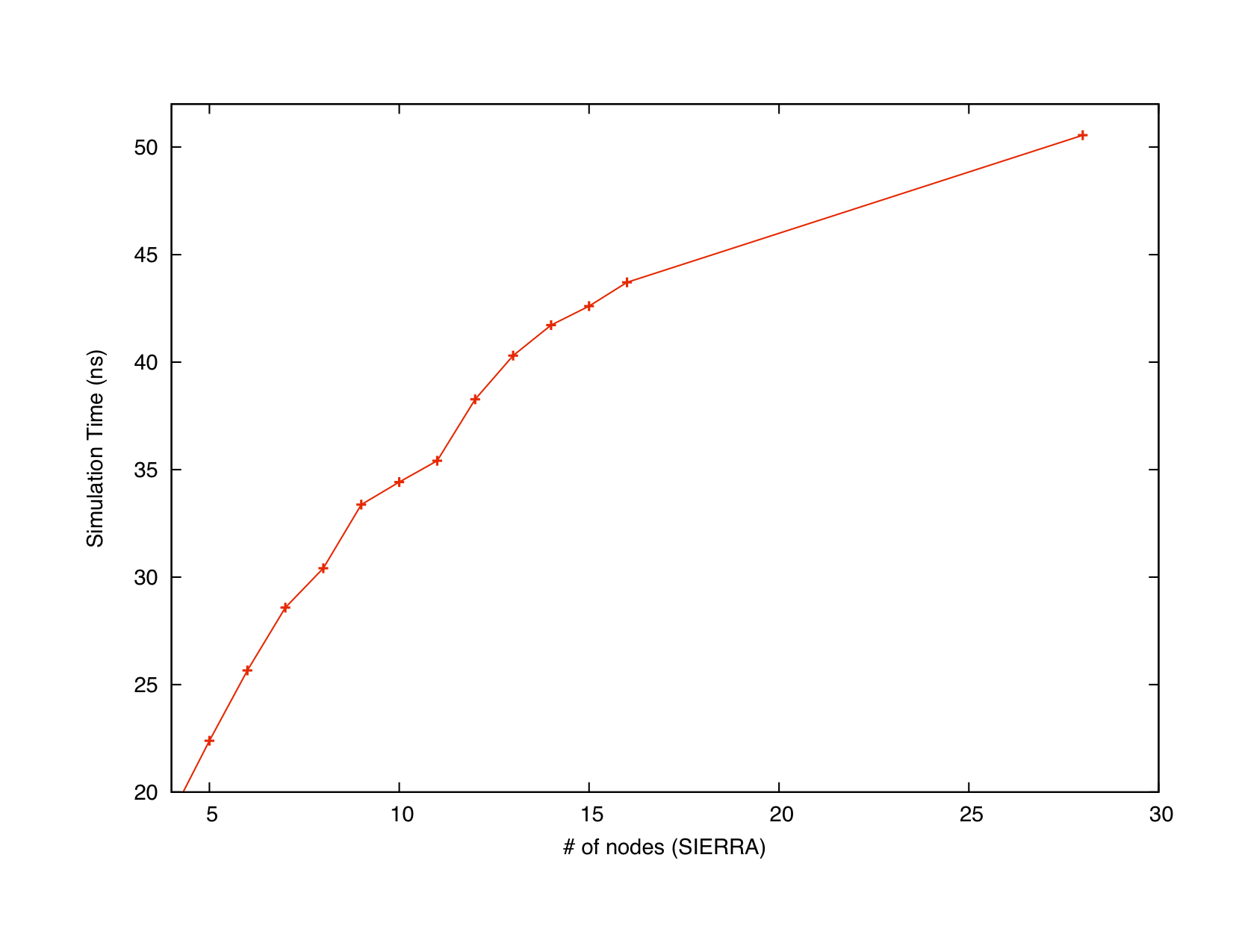


**Chart S1.** Scaling performance of AMBER PMEMD for NPT simulation on a test system of 50,000 atoms (including explicit TIP3P waters).

**IV. References**

1. Sansen S, Yano JK, Reynald RL, Schoch GA, Griffin KJ, et al. (2007) Adaptations for the oxidation of polycyclic aromatic hydrocarbons exhibited by the structure of human P450 1A2. Journal of Biological Chemistry 282: 14348-14355.

2. Yano JK, Denton TT, Cerny MA, Zhang XD, Johnson EF, et al. (2006) Synthetic inhibitors of cytochrome P-450 2A6: Inhibitory activity, difference spectra, mechanism of inhibition, and protein cocrystallization. Journal of Medicinal Chemistry 49: 6987-7001.

3. Yano JK, Hsu MH, Griffin KJ, Stout CD, Johnson EF (2005) Structures of human microsomal cytochrome P450 2A6 complexed with coumarin and methoxsalen. Nature Structural & Molecular Biology 12: 822-823.

4. Wester MR, Yano JK, Schoch GA, Yang C, Griffin KJ, et al. (2004) The structure of human cytochrome P4502C9 complexed with flurbiprofen at 2.0-angstrom resolution. Journal of Biological Chemistry 279: 35630-35637.

5. Williams PA, Cosme J, Ward A, Angova HC, Vinkovic DM, et al. (2003) Crystal structure of human cytochrome P4502C9 with bound warfarin. Nature 424: 464-468.

6. Porubsky PR, Meneely KM, Scott EE (2008) Structures of Human Cytochrome P-450 2E1 INSIGHTS INTO THE BINDING OF INHIBITORS AND BOTH SMALL MOLECULAR WEIGHT AND FATTY ACID SUBSTRATES. Journal of Biological Chemistry 283: 33698-33707.

7. DeVore NM, Meneely KM, Bart AG, Stephens ES, Battaile KP, et al. (2012) Structural comparison of cytochromes P450 2A6, 2A13, and 2E1 with pilocarpine. Febs Journal 279: 1621-1631.

8. Sevrioukova IF, Poulos TL (2010) Structure and mechanism of the complex between cytochrome P4503A4 and ritonavir. Proceedings of the National Academy of Sciences of the United States of America 107: 18422-18427.

9. Yano JK, Wester MR, Schoch GA, Griffin KJ, Stout CD, et al. (2004) The structure of human microsomal cytochrome P450 3A4 determined by X-ray crystallography to 2.05-angstrom resolution. Journal of Biological Chemistry 279: 38091-38094.

10. Sevrioukova IF, Poulos TL (2012) Structural and Mechanistic Insights into the Interaction of Cytochrome P4503A4 with Bromoergocryptine, a Type I Ligand. Journal of Biological Chemistry 287: 3510-3517.

11. Chen WQ, Koenigs LL, Thompson SJ, Peter RM, Pettie AE, et al. (1998) Oxidation of acetaminophen to its toxic quinone imine and nontoxic catechol metabolites by baculovirus-expressed and purified human cytochromes P450 2E1 and 2A6. Chemical Research in Toxicology 11: 295-301.

12. Hazai E, Vereczkey L, Monostory K (2002) Reduction of toxic metabolite formation of acetaminophen. Biochemical and Biophysical Research Communications 291: 1089-1094.

13. Zaher H, Buters JTM, Ward JM, Bruno MK, Lucas AM, et al. (1998) Protection against acetaminophen toxicity in CYP1A2 and CYP2E1 double-null mice. Toxicology and Applied Pharmacology 152: 193-199.

14. Snawder JE, Roe AL, Benson RW, Roberts DW (1994) Loss of Cyp2e1 and Cyp1a2 Activity as a Function of Acetaminophen Dose - Relation to Toxicity. Biochemical and Biophysical Research Communications 203: 532-539.

15. Sarich T, Kalhorn T, AlSayegh F, Adams S, Slattery J, et al. (1997) The effect of omeprazole pretreatment on acetaminophen metabolism in rapid and slow metabolizers of S-mephenytoin. Clinical Pharmacology & Therapeutics 62: 21-28.

16. Patten CJ, Thomas PE, Guy RL, Lee MJ, Gonzalez FJ, et al. (1993) Cytochrome-P450 Enzymes Involved in Acetaminophen Activation by Rat and Human Liver-Microsomes and Their Kinetics. Chemical Research in Toxicology 6: 511-518.

17. Dong HJ, Haining RL, Thummel KE, Rettie AE, Nelson SD (2000) Involvement of human cytochrome P450 2D6 in the bioactivation of acetaminophen. Drug Metabolism and Disposition 28: 1397-1400.

18. Rendic S (2002) Summary of information on human CYP enzymes: Human P450 metabolism data. Drug Metabolism Reviews 34: 83-448.

19. Li DM, Wang Y, Han KL (2012) Recent density functional theory model calculations of drug metabolism by cytochrome P450. Coordination Chemistry Reviews 256: 1137-1150.

20. Williams PA, Cosme J, Vinkovic DM, Ward A, Angove HC, et al. (2004) Crystal structures of human cytochrome P450 3A4 bound to metyrapone and progesterone. Science 305: 683-686.

21. Mosher CM, Hummel MA, Tracy TS, Rettie AE (2008) Functional Analysis of Phenylalanine Residues in the Active Site of Cytochrome P450 2C9. Biochemistry 47: 11725-11734.

22. Wang Y, Chen H, Makino M, Shiro Y, Nagano S, et al. (2009) Theoretical and Experimental Studies of the Conversion of Chromopyrrolic Acid to an Antitumor Derivative by Cytochrome P450 StaP: The Catalytic Role of Water Molecules. Journal of the American Chemical Society 131: 6748-6762.

23. Gumbart JC, Roux B, Chipot C (2013) Standard Binding Free Energies from Computer Simulations: What Is the Best Strategy? Journal of Chemical Theory and Computation 9: 794-802.
